# Implantation of *Bacillus pseudomycoides* Chromate Transporter Increases Chromate Tolerance in *Bacillus subtilis*

**DOI:** 10.3389/fmicb.2022.842623

**Published:** 2022-03-07

**Authors:** Zuzana Chromiková, Romana Kalianková Chovanová, Dragana Tamindžija, Barbora Bártová, Dragan Radnović, Rizlan Bernier-Latmani, Imrich Barák

**Affiliations:** ^1^Department of Microbial Genetics, Institute of Molecular Biology, Slovak Academy of Sciences, Bratislava, Slovakia; ^2^Department of Chemistry, Faculty of Sciences, Biochemistry and Environmental Protection, Novi Sad, Serbia; ^3^Department of Biology and Ecology, Faculty of Sciences, University of Novi Sad, Novi Sad, Serbia; ^4^Environmental Microbiology Laboratory, Ecole Polytechnique Fédérale de Lausanne, Lausanne, Switzerland

**Keywords:** chromate tolerance, chromate efflux pump, *B. subtilis*, *B. pseudomycoides*, bioremediation

## Abstract

Chromium of anthropogenic origin contaminates the environment worldwide. The toxicity of chromium, a group I human carcinogen, is greatest when it is in a hexavalent oxidation state, Cr(VI). Cr(VI) is actively transported into the cell, triggering oxidative damage intracellularly. Due to the abundance of unspecific intracellular reductants, any microbial species is capable of bio-transformation of toxic Cr(VI) to innocuous Cr(III), however, this process is often lethal. Only some bacterial species are capable of sustaining the vegetative growth in the presence of a high concentration of Cr(VI) and thus operate as self-sustainable bioremediation agents. One of the successful microbial Cr(VI) detoxification strategies is the activation of chromate efflux pumps. This work describes transplantation of the chromate efflux pump from the potentially pathogenic but highly Cr resistant *Bacillus pseudomycoides* environmental strain into non-pathogenic but only transiently Cr tolerant *Bacillus subtilis* strain. In our study, we compared the two *Bacillus* spp. strains harboring evolutionarily diverged chromate efflux proteins. We have found that individual cells of the Cr-resistant *B. pseudomycoides* environmental strain accumulate less Cr than the cells of *B. subtilis* strain. Further, we found that survival of the *B. subtilis* strain during the Cr stress can be increased by the introduction of the chromate transporter from the Cr resistant environmental strain into its genome. Additionally, the expression of *B. pseudomycoides* chromate transporter ChrA in *B. subtilis* seems to be activated by the presence of chromate, hinting at versatility of Cr-efflux proteins. This study outlines the future direction for increasing the Cr-tolerance of non-pathogenic species and safe bioremediation using soil bacteria.

## Introduction

Chromium, as a transition metal, is found in a variety of oxidation states, out of which the most stable are trivalent Cr(III) and hexavalent Cr(VI). Cr(III) is mostly present in minerals in the form of [Cr(OH)_3_] or hydrated oxides (Ehrlich, 2002), which due to their inability to cross cell membranes are considered to be of low toxicity. In contrast, Cr(VI) is soluble in water, therefore highly mobile and extremely toxic (Cervantes et al., 2001). Chromium speciation and mobility in a given environment depend on its biogeochemical cycling. Due to the structural similarity to the sulfate anion SO_4_^2–^, Cr(VI) is actively transported across cell membranes (Cervantes and Campos-García, 2007). The toxicity of Cr(VI) is caused by the induction of massive oxidative stress in the cell, triggered by the generation of the highly reactive intermediates Cr(V) and Cr(IV) during the intracellular reduction of Cr(VI). These tend to re-oxidize back to Cr(VI), or give rise to reactive oxygen species (ROS) after reaction with intracellular oxygen. Generated ROS target cytoplasmic membranes, proteins and DNA, which eventually leads to the inhibition of vital cellular processes (Kanmani et al., 2012).

Prokaryotes employ several strategies to combat chromate stress. Some of them are not specific to chromate exposure but hinder the entry of Cr(VI) into the cell, such as passive sequestration of chromium ions by the cell surface, spores or intracellular matrix (Fein et al., 2002; Nancharaiah et al., 2010; Sturm et al., 2018). Other response mechanisms are chromium-specific and include the shutdown of sulfate ABC transporters responsible for the CrO_4_^2–^ entry (Viti et al., 2014) or the intra- or extracellular enzymatic reduction of Cr(VI) (Ramírez-Díaz et al., 2008). Intracellular chromate-specific reductases minimize the number of electron transfers necessary for reduction of Cr(VI) to Cr(III), thus diminishing the amount of ROS generated and the level of oxidative stress (Ackerley et al., 2004). Upon Cr(VI) entry, some bacteria activate chromate efflux pumps, which are responsible for its extrusion. The last line of defense for the cell is to fight off oxidative stress by activating ROS-scavenging enzymes as well as DNA repair mechanisms (Hu et al., 2005; Ackerley et al., 2006; Cervantes and Campos-García, 2007).

In the present study, we focused on the contribution of chromate-specific efflux pumps to the overall microbial chromate tolerance. Chromate efflux determinant was first identified as a gene present on a conjugal plasmid of a *Pseudomonas aeruginosa* clinical strain, responsible for conferring the Cr resistance (Cervantes et al., 1990). After the increased availability of bacterial genomes sequencing, multiple homologs of ChrA were identified across the bacterial kingdom (Nies et al., 1998). The presence of chromate transporter genes in bacterial genomes has been linked to their elevated resistance to chromate stress. Chromate transporter proteins are presumed to act as chemiosmotic pumps, extruding chromate from the cell cytoplasm using the proton motive force (Nies, 2003; Díaz-Pérez et al., 2007). The range of chromate concentrations to which bacteria harboring chromate transporter genes are resistant varies from sub-millimolar (0.35 mM) to close to molar (200 mM) (Juhnke et al., 2002; Henne et al., 2009; Monsieurs et al., 2011), indicating that the sole presence of a chromate transporter is generally not a sufficient prerequisite for high chromate resistance. A plausible explanation for the variation in chromate tolerance level amongst chromate transporter-harboring species might be the diversity of the chromate ion transporter (CHR) superfamily, to which these proteins belong. The CHR protein superfamily stretches across archaea, bacteria and fungi domains and is divided, based on the length of the actual transporter protein, in LCHR (long-chain chromate transporters) and SCHR (short-chain chromate transporters) families (Díaz-Pérez et al., 2007). The SCHR family comprises mono-domain proteins of around 200 aa in length, while the LCHR family consists of bi-domain proteins 400 aa long. Interestingly, certain strains encode a combination of mono- and bi-domain chromate transporters in their genomes, located either on the chromosome or a plasmid, with different contributions to overall chromate resistance, as in the case for *Burkholderia xenovorans* (Reyes-Gallegos et al., 2016). All chromate transporters typically possess multiple transmembrane segments (TMS). Very little is known about Cr efflux regulation, as only two subfamilies of LCHR family (LCHR2 and LCHR5) and one subfamily of SCHR (SCHR1) are known to express regulatory genes together with the chromate transporter (Díaz-Pérez et al., 2007; Aguilar-Barajas et al., 2013). These regulatory genes differ in number as well as in mode of action across genera, species, or even individual strains (Branco et al., 2008; Henne et al., 2009; He et al., 2010; Aguilar-Barajas et al., 2013; Klonowska et al., 2020).

In a previous study, we isolated a highly Cr-resistant environmental strain, *Bacillus pseudomycoides* NCr1a (Tamindžija et al., 2019). This strain exhibited resistance to 8 mM Cr(VI) in minimal M9 medium and, thus, was far more resistant to Cr(VI) than any of the other environmental strains isolated in that study (Tamindžija et al., 2019). Here, using scanning transmission electron microscopy-energy dispersive spectroscopy (STEM-EDS), we mapped and quantified chromium accumulation by individual cells of the Cr-resistant environmental strain and compared it to that of the Cr-tolerant, but not resistant *B. subtilis* PY79. We found that the cells of Cr-resistant environmental strain NCr1a accumulated less chromium than the moderately Cr-tolerant *B. subtilis*. Two of the hitherto identified Cr-specific response mechanisms are known to oppose the accumulation of Cr in the cell. The first one is the shutdown of sulfate ABC transporters, which facilitate the unspecific active import of Cr(VI) into the cell. Mutagenesis of these transporter proteins is presumed as an underlying mechanism for this type of defense (Viti et al., 2014; Xiao et al., 2020). However, in case of sudden Cr exposure, the shutdown of ABC transporters is not immediately feasible, since it is reported to result from the powerful, but rather slow bacterial acclimation process lasting several days (Xiao et al., 2020). Since the strains analyzed in this study were not acclimated prior exposure to Cr(VI), we focused on the other Cr-specific response mechanism opposing the accumulation of Cr inside cells, the Cr efflux. The two strains compared harbor different types of chromate transporters, thus we hypothesized that the chromate efflux efficacy of these proteins varied and could explain the differences in the immediate response of the analyzed strains to Cr(VI). We concentrated on the putative chromate transporter from the Cr-resitant environmental *B. pseudomycoides* NCr1a, which is homologous to the ChrA of *B. cereus*. Introduction of the gene encoding ChrA from NCr1a to the genome of *B. subtillis* increased the chromate tolerance of the engineered strain. The presence of ChrA also rescued the Cr-sensitive phenotype of *B. subtilis* mutant strain lacking the operon harboring its innate chromate efflux pump, *ywrBA*. To assess the level of *chrA* expression in the heterologous system of *B. subtilis*, we monitored the GFP signal of the GFP-ChrA fusion placed under control of its original P_chrA_ promoter. We found that the P_chrA_ promoter is recognized in *B. subtilis*, and that the expression of GFP-ChrA under its control seems to be partially dependent on the concentration of chromium in the environment.

## Materials and Methods

### Construction of Strains

The *B. pseudomycoides* sp., *B. subtilis* and *E. coli* strains, plasmids and sequences of oligonucleotides used in this study are available in Supplementary Tables 1–3. Plasmids were constructed using standard molecular biology methods (Ausubel et al., 2003) and amplified in *E. coli* MM294 or DH5α cells. PCR fragments were amplified from *B. pseudomycoides* NCr1a (Tamindžija et al., 2019) or *B. subtilis* PY79 chromosomal DNA. The nucleotide sequence of *chrA* cds and its upstream reagion was extracted from NCr1a genomic DNA using primers complementary to *B. pseudomycoides* DSM 12442 type strain genomic sequence (NCBI Reference Sequence: NZ_CM000745.1; locus tag “bpmyx0001_46360”; protein_id = “EEM14515.1”). The PCR fragment was sequenced (Eurofins Genomics LLC). The acquired sequence was translated into a protein using Vector NTI software (Invitrogen) and compared against the protein database using NCBI Standard Protein Blast tool, to confirm we extracted the sequence encoding the putative chromate transporter. The translated sequence was also compared to sequences of other previously characterized chromate transporters such as ChrA from *B. cereus* SJ1 (NCBI Reference Sequence: NZ_CP072774.1; protein_id = “WP_048538372.1”; “GeneID:67509833”), *Lysinibacillus fusiformis* ZC1 (NCBI reference Sequence NZ_ADJR01000017.1; locus_tag = “BFZC 1_RS06765”; protein_id = “WP_192807144.1”), *Ochrobactrum triticii* TnOtChr (GenBank: EF469735.1; protein_id = “ABO7 0325.1”), *Shewanella oneidensis* MR-1 (NCBI Reference Sequence: NC_004347.2; locus_tag = “SO_RS04615”; protein _id = “WP_011071255.1”), *Shewanella* sp. ANA-3 (NCBI Reference Sequence: NC_008577.1; locus_tag = “SHEWANA 3_RS17310”; protein_id = “WP_041412773.1”), (*Pseudomonas aeruginosa* pUM505 (NCBI Reference Sequence: NC_01613 8.1; locus_tag = “HS786_RS00455”; protein_id = “WP_0031 17270.1”).

To construct the *B. subtilis* strain P_chrA_-*chrA*, a 1,414 bp region of NCr1a chromosomal DNA containing the *chrA* gene together with 317 bp of its upstream region was amplified using chrABP_Up_AccF and chrABP_STOP_BamR primers. This fragment was then cloned into Acc651 and *Bam*HI sites of integrative vector pSG1729. The presence of the insert in the resulting plasmid was verified by restriction and sequence analysis. Resulting pSG17PchrA-chrA plasmid was used for transformation of *B. subtilis* PY79 as described previously (Feucht and Lewis, 2001). The integration of P_chrA_-*chrA* into *amyE* locus was confirmed by PCR. To construct *B. subtilis* Δ*ywrBA* strain, we used the vector pMAD designed for allelic replacement in multiple Gram positive hosts in a way that was described before (Arnaud et al., 2004). Shortly, we used chromosomal DNA of *B. subtilis* PY79 as a template as well as primers U1000_ywrB_BamF6 and U1000_ywrB_SalR2 to amplify region 936 bp upstream of *ywrB* gene and cloned the PCR fragment into *Bam*HI, *Sal*I restriction sites of pMAD. Similarly, primers D1000_ywrA_SalF2 and D1000_ywrA_EcoRIR were used to amplify region 948 bp downstream of *ywrA* gene and the PCR fragment was cloned into *Sal*I, *Eco*RI restriction sites of pMAD. Primers Kan_new_SalF and Kan_new_SalR2 were used to amplify the sequence of the Kanamycin marker fom pUK19 plasmid template. The PCR fragment carrying promoter together with the coding sequence of kanamycin resistance gene was cloned into *Sal*I restriction site. The final construct, pMAD Up_*ywrB*:*kan*:down_*ywrA* was verified by restriction and sequence analysis. To replace *ywrBA* operon in *B. subtilis* PY79 with the kanamycin resistance cassette, the construct pMAD Up_*ywrB*:*kan*:down_*ywrA* was introduced into *B. subtilis* cells by electroporation (conditions listed in section below). The transformation mixture was plated on Erm and X-gal containing plates and incubated overnight at 37°C. Transformants that appeared blue on X-gal media were the ones containing pMAD construct in the cell as a shuttle. These were pooled and re-streaked onto LB agar media containing X-gal, Erm and Kan, followed by incubation overnight. Liquid LB media containing Erm, Kan were inoculated by blue transformants and incubated overnight at 37^°^C with shaking. The overnight culture was used for inoculation of fresh LB to starting OD600 0.4, which was followed by a 2-h incubation at 37°C with shaking. After that, the temperature was raised to a non-permissive 40°C to promote plasmid integration into the chromosome. The culture was incubated at 40°C for 6 h. Eventually, cultures were serially diluted and plated onto LB agar plates containing X gal and Kanamycin. Transformants that appeared white on Kan, X gal containing plates were tested for Erm sensitivity. Clones which were white on X-gal, resistant to kanamycin but sensitive for erythromycin were the ones that completed allelic replacement and lost the plasmid, leaving kanamycin cassette at the locus of former *ywrBA* operon. The lack of *ywrBA* operon as well as the presence of kanamycin cassette in the resulting strain was confirmed by PCR and sequence analysis.

To construct the *B. subtilis* P_chrA_-*chrA* Δ*ywrBA* strain, the aforementioned plasmid pSG17P_chrA_-*chrA* was used to introduce *chrA* coding sequence under the control of its promoter into *amyE* locus of Δ*ywrBA* strain. The resulting strain was resistant to kanamycin and chloramphenicol. The presence of *amyE*:P_chrA_-*chrA* chloramphenicol cassette was verified by PCR using primers Up_chrABP_Prom_AccF, Up_chrABP_Prom_AccR.

To create *B. subtilis* strain P_chrA_-*gfp-chrA*, the upstream region of *chrA* gene was amplified using primers Up_chrABP_Prom_AccF, Up_chrABP_Prom_AccR and chromosomal DNA of NCr1a as a template. The product of 315 bp was cloned into Acc651 restriction site of pSG1729, upstream of *gfp* gene present in the plasmid (Feucht and Lewis, 2001), creating the intermediate pSG17P_chrA_-*gfp*, which was sequenced to confirm the correct orientation and sequence of the upstream *chrA* promoter containing region. Subsequently, *chrA* coding sequence was amplified using primers chrABP_GFP_EcoF, chrABP_GFP_EcoR and chromosomal DNA of NCr1a as a template. The product of 1,095 bp, *chrA* cds was cloned into *Eco*RI restriction site of pSG17PchrA-*gfp* intermediate, to create translation fusion with *gfp* positioned upstream. The orientation of the insert was verified by restriction analysis and resulting plasmid P_chrA_-*gfp-chrA* was sequenced prior introduction into *B. subtilis* PY79 *amyE* locus.

### Electroporation of *Bacillus subtilis*

The procedure of preparation of electro-competent cells of *B. subtilis*, as well as conditions for electroporation, was combined according to previously published works (Lu et al., 2012; Zhang et al., 2015). Briefly, electro-competent cells were prepared by incubation of B. subtilis culture in LSBP media (Zhang et al., 2015) at 37°C until OD600 0.85–1.0 was reached. Subsequently, the culture was cooled on ice for 10 min, centrifuged at 4°C and washed 4 times with the electroporation medium (Lu et al., 2012). The cells were resuspended in an appropriate amount of electroporation medium and stored at –70°C. For electroporation, 60 μl of the competent cells were mixed with 10–200 ng/μl plasmid in an ice-cold electroporation cuvette (1 mm gap, Bio-Rad, United States). After incubation for 5–10 min on ice, the mixture was pulsed using a pulser apparatus (Bio-Rad, United States) with parameters 25 μF, 200 Ω, 15 kv/cm. Subsequently, 1 ml of recovery medium was added to the transformation mixture and cells were incubated for 3 h at 37°C with shaking. Eventually, cells were gently pelleted by centrifugation at room temperature, the pellet was resuspended in 100 μl of the supernatant and suspension was plated on selective LB agar media, followed by overnight incubation at 37°C.

### Bacterial Cultures

For *B. subtilis* cultivation, a fresh single colony from overnight LBA plate was spread-inoculated onto LB plate and incubated overnight at 30°C. *B. pseudomycoides* NCr1a cultivation was started by inoculation of a fresh single colony to a liquid LB followed by incubation overnight at 28°C with shaking (150 rpm). Then, the overnight cultures were harvested and re-suspended in 1X Phosphate Buffer Saline (PBS), pH 7.4 (0.137 M NaCl; 0.0027 M KCl; 0.01M Na_2_HPO_4_; 0.0018 M KH_2_PO_4_), washed 2 times, and the OD600 was set to 4.0 in 1X PBS. The dense cell suspension was used for inoculation of M9 media (Atlas, 2010), supplemented with L-Tryptophane to a final concentration of 20 μg⋅ml^–1^; Thiamin to a final concentration of 1 mM; and Cas-amino acids to final concentration 0.01%, where necessary supplemented with K_2_CrO_4_ as Cr(VI). The starting OD600 was set to 0.15. Samples were cultivated at 37°C (*B. subtilis*) or 28°C (*B. pseudomycoides* NCr1a) with shaking (150 rpm) and their growth was monitored by measuring OD600 in 1 h intervals.

#### Preparation of Bacterial Samples for Scanning Transmission Electron Microscopy With Energy Dispersive Spectroscopy

The strains were cultivated as described above. OD600 was measured each hour in exponential phase, and every 6 h at later stages of growth. In one set of experiments, the Cr-tolerant NCr1a and the Cr-sensitive *B. subtilis* PY9 were grown in minimal media containing 1 mM Cr(VI). Cells of both strains were harvested at either the exponential or stationary phase of growth. In the other set of experiments, both strains were grown in minimal media without Cr(VI) until reaching the exponential phase. At this point, the cells of both strains were harvested and divided into two groups. The first group of cells was immediately exposed to 1 mM Cr(VI) for 1 additional hour. The second group of cells was treated by sub-inhibitory 10 μM concentration of Cr(VI) for 1 h to induce Cr resistance. Subsequently, these cells were washed and exposed to 1 mM Cr(VI) in PBS for an additional hour. After the harvest, cells were fixed with 2.5% glutaraldehyde buffer for 30 min, washed, and stored in PBS. Prior to STEM-EDS analysis the suspension was washed 3 times in distilled water to remove salt residues from PBS which could affect the analysis. *B. subtilis* cultures grown with Cr(VI) had to be concentrated because their growth was very limited.

### Scanning Transmission Electron Microscopy With Energy Dispersive Spectroscopy Analysis

Scanning Transmission Electron Microscopy with energy dispersive spectroscopy (STEM-EDS) was used to obtain elemental composition maps and to compare elemental content in the two examined bacterial strains’ cells. Elemental content was reported in atomic percentages (at%), which is the percentage of one kind of atom relative to the total number of atoms. Data were acquired by using an X-ray EDS system (Esprit/Quantax Bruker) in STEM mode of a microscope FEI Tecnai Osiris [200 kV X-FEG field emission gun, X-ray detector (Super-X) with 4 mm × 30 mm windowless SDD diodes and 0.9 sr collection angle] at following conditions: 200 kV, 1.2 nA beam current as was described previously (Jamroskovic et al., 2016). Quantitative EDS analysis was carried out using the Cliff–Lorimer standard-less method with thickness correction using K-series. The physical Bremsstrahlung background was calculated based on the sample composition. Some elements such as Cu originating from the Cu grid were removed from quantification after the deconvolution procedure in the quantification process. Elemental concentrations in atomic% and net counts (signal above background) were derived from deconvoluted line intensities within a 95% confidence level. The processing time and acquisition rates were adapted to get the most accurate data for a specific element such as Ca, P, Cr, and Mn. The experimental spectra were collected with no pile-up artifacts. A correction for specimen drift was applied during acquisition to improve elemental mapping accuracy.

### Estimation of Strain Cr(VI) Tolerance

Strains were cultivated as described above, in M9 media supplemented with ascending concentrations of K_2_CrO_4_ to a final concentration of Cr(VI) of 0 mM, 0.1, 0.3, 0.5, and 1.0 mM. The growth of strains was monitored by measuring OD600 in 30 min intervals. During each experiment, each strain was grown in a triplicate for each concentration of Cr(VI) tested. The growth curve presented for each strain and concentration of Cr(VI) is a result of median calculated from OD600 values gained in four independent experiments.

#### Estimation of Doubling Time (T_d_)

Bacterial growth is described by sigmoid growth function, defined by the three parameters corresponding to three characteristic phases of growth, the lag phase, the log phase and the stationary phase (Baranyi and Roberts, 1995). From these, the log phase can be fitted to an exponential model, which is the basis for the calculation of a doubling time (T_d_) for bacterial strain in given conditions (Maier, 2009). The log phase of growth curves of analyzed strains was fitted to an exponential function with different degrees of accuracy and resulting doubling times were compared (Supplementary Figure 1).

### Estimation of Strain Fitness After Treatment by Cr(VI)

Strains were cultivated as described above. After 6 h of exposure to Cr(VI), decimal serial dilutions of the culture grown without Cr as well as of cultures exposed to Cr(VI) were prepared. The selected dilutions were plated onto M9 agar plates in duplicates. The number of CFU for each concentration of Cr(VI) was assessed for each strain, and their viability was expressed as a percentage of CFU counts of the same strain cultivated without Cr. The experiment was performed 3 times in an independent manner.

### Fluorescence Microscopy

In all experiments, the strains to be analyzed were grown as described above. For all strains grown in the presence of Cr(VI), cultures grown without Cr(VI) served as negative controls. After the staining with fluorescence dyes, 1 ml of sample was centrifuged at RT, 5,000 rpm, 3 min. Except for *Propidium iodide* staining, the samples were washed 3 times by 1 × PBS and subsequently were 100 × concentrated. 0.5 μl of the sample was put on poly-lysine-covered slide and subjected to fluorescence microscopy.

For *CellROX*™ *Deep Red* (Thermo Fisher Scientific) staining, strains NCr1a and *B. subtilis PY79* were grown in M9 media supplemented with 1 mM K_2_CrO_4_ [Cr(VI)] for 6 h. Thirty minutes prior harvest, the *CellROX™ Deep Red* reagent was added to 5 ml of culture to reach a final concentration of 5 μM. Images were obtained using the Texas Red filter.

For Live/Dead staining, *Rhodamin 123* and Propidium Iodide (Sigma Aldrich) were used. In the case of *Rhodamin 123* staining, *B. subtilis* strains were grown in M9 media supplemented with 1 mM K_2_CrO_4_ [Cr(VI)] for 6 h. Twenty minutes prior the harvest, *Rhodamin 123* was added to cell cultures to a final concentration 0.1μg⋅ml^–1^. Images were obtained using the FITC filter. In the case of *Propidium Iodide* (Sigma Aldrich) staining *B. subtilis* strains were grown in M9 media supplemented with 1 mM K_2_CrO_4_ [Cr(VI)] for 6 h. Then PI was added to 1 ml of the sample to reach the final concentration of 1 μg⋅ml^–1^ and was left to incubate for 5 min at the RT, followed by centrifugation as described above. No washing by PBS was involved, as this resulted in de-staining. Images were obtained using the DS Red Filter.

For *mGFP-ChrA* fluorescence microscopy, P_chrA_-GFP-ChrA *B. subtilis* strain was grown as described above in M9 media supplemented with ascending concentrations of K_2_CrO_4_ [Cr(VI)] for 6 h. Images were obtained using the FITC filter.

Images were acquired using an Olympus BX63 microscope equipped with a sCMOS Zyla-4.2P camera (Andor, Oxford Instruments, Belfast, United Kingdom). Olympus CellP imaging software and ImageJ v1.53f software were used for image acquisition and analysis.

### Western Blot Analysis

The 200 ml cultures were started as usual, in M9 media supplemented with ascending concentrations of K_2_CrO_4_, from 0 to 0.5 mM. Pellets were frost in liquid nitrogen and stored at –80°C. Prior use, pellets were concentrated in 2 ml of the Solubilization buffer [20 mM TrisHCl (pH 8), 150 mM NaCl, 1 mM AEBSF] to OD600 = 20. To each suspension, the lysozyme was added to a final concentration of 1 mg/ml and consequently, the cells were lysed by sonication (15.000 kHz, 20 times for 9 s with a 1-min break and constant cooling). 50 μl of the “TOTAL” fraction was collected for each sample, as were fractions “SOLUBLE” and “PELET” following centrifugation at 30,000 rpm; 6°C for 30 min. All samples were mixed with 2 × Sample Laemmli Buffer (4% SDS, 20% glycerol, 10% 2-mercaptoethanol, 0.004% bromphenol blue and 0.125 M Tris HCl, pH. 6.8) and boiled for 3 min before loading 20 μl of each sample to 12% SDS gel. The proteins were blotted onto nitrocellulose membrane by semi-dry transfer (Biometra Fast Blot, Analytik Jena, GmbH) at 205 mA, 10 W, 12 V, for 1 h. After washing the membrane with TBST buffer (20 mM Tris, 150 mM NaCl, 0.1% (w/v) Tween^®^ 20) and blocking with 5% (w/v) milk in TBST, the membrane was cut in half according to the molecular weight protein standard PageRuler™ Plus Prestained (Thermo Fisher Scientific) bands. The upper half of the membrane containing GFP-ChrA fusion protein (70 kDa) was incubated with the monoclonal mouse anti−GFP antibody [Anti-GFP antibody (9F9.F9) (ab1218); Abcam, Cambridge, CB2 0AX, United Kingdom] diluted to 1:1,000 in 5% (w/v) milk in TBST overnight, at 4°C. The other half of the membrane, containing major RNA-polymerase subunit SigA a member of Sig70 protein family (43 kDa) was incubated with the monoclonal mouse anti-Sig70 antibody [(ab12088) Anti-RNA polymerase sigma 70 antibody (2G10); Abcam, Cambridge, CB2 0AX, United Kingdom] diluted to 1:1,000 in 5% (w/v) milk in TBST for 1 h, RT, as described previously (Strickland et al., 1988; Jovanovich et al., 1989; Breyer et al., 1997). Subsequently, the membranes were incubated with anti-mouse HRP conjugated antibody (Promega) diluted 1:2,500 in 5% (w/v) milk in TBST for 1 h, RT. The Western blot analysis was performed with a modified ECL peroxidase detection kit (Bio-Rad). The signal was developed using RTG films, scanned and subjected to ImageJ analysis. For quantification of Western blots, the adaptation of the Luke Miller protocol was used.^1^ The representative image shows the GFP and SigA signal coming from the same sample for each concentration of Cr(VI) tested. All samples were run on the same gel, and blotted to the same membrane. The GFP and SigA signal were developed separately. Altogether, in this study we present the results of three independent Western blots, where the average SigA signals were synchronized so that the GFP-ChrA signals could be compared and plotted together with standard errors. In this process, the average values of the percentage of peaks for the SigA signal in one Western blot were normalized to that of the second, and to that of the third Western blot. All profile plot percentage values were then recalculated with this normalization coefficient.

### Localization and Quantification of CellRox™ Deep Red Signal

Images obtained by CellRox™ Deep Red fluorescence microscopy were saved with optimal settings as TIF stacked images by CellP imaging software directly during the experiment. For analysis in ImageJ,^2^ stacked images were set to the same values of brightness, contrast and intensity, and split to a phase-contrast image and fluorescence signal image. All phase-contrast images were adjusted to the same values of threshold, rendered binary and processed with watershed function to discern cells in chains. Such image was used for counting of particles (cells) with particle size characteristics being 250− infinity pixel^2, circularity 0.00–0.8 to fit rod-like *Bacillus* sp. cells. By this approach total number of cells in each image was obtained. Phase-bright or translucent cells were considered to be dead and were counted manually in each image hence the number of live cells was estimated by subtraction of the number of dead cells from the number of all (total) cells. The image with the fluorescence signal was inverted, and after subtraction of background by Rolling Ball function was processed by Auto Local Thresholding. Up-coming measurements were set to analyze the area taken up by the signal, integrated density of the signal and minimal and maximal mean value of the signal. Selections, where these characteristics were to be analyzed, were defined by the outlines of the signal foci. These were obtained after particle analysis of a binary version of fluorescence image, where the particle was defined as being of size 50-infinity pixel^2, with 0.00–1.00 circularity, to fit the above-noise size and any shape of fluorescence signal. Outlines of fluorescence signal foci were saved as ROIs for each image. Particle analysis set for assessment of the signal area, integrated density and maximal and minimal mean value provided these data for each ROI. Per each type of sample [PY79_0 mM Cr(VI); PY79_1 mM Cr(VI); NCr1a_0 mM Cr(VI); NCr1a_1 mM Cr(VI)] six images were analyzed in total, together representing over 3,000 cells. Overlay of fluorescence signal derived ROIs with the phase-contrast image enabled exclusion of signals coming from dead cells or surrounding media. From these data, the average of integrated density of the fluorescence signal was estimated for each type of sample and was taken as the means to express the overall signal intensity. Similarly, the average of the signal integrated density in the live cells was estimated as well as the number of live cells displaying the fluorescence signal.

## Results

### Accumulation of Cr in Cells of *Bacillus pseudomycoides* NCr1a and *Bacillus subtilis* PY79

Bacteria employ several mechanisms to minimize the impact of Cr on their vital processes. After the limits of these defenses are met, the build-up of intracellular Cr species intracellularly is inevitable.

Using the STEM-EDS technique that allows quantitative mapping of chromium in individual cells, we compared the cellular accumulation of chromium for two species with differing Cr tolerance. The minimal inhibitory concentration of Cr has been previously estimated for both strains (Tamindžija et al., 2019).

**FIGURE 1:**
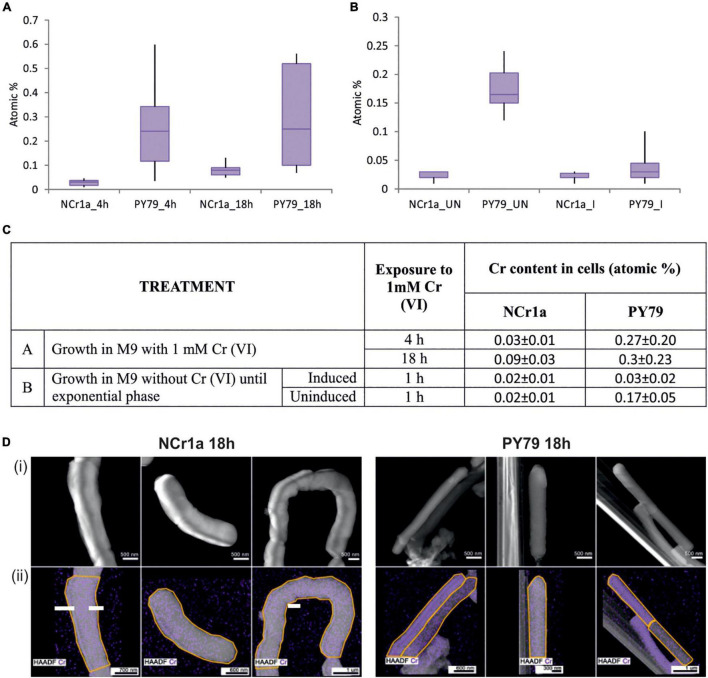
Accumulation of Cr by cells after growth with 1 mM Cr(VI). **(A,B)** Box plot diagrams describe the range and distribution of Cr content between cell categories, using quartiles. At least 10 different cells were analyzed for each group. **(A)** The accumulation of Cr by cells of Cr-tolerant NCr1a strain and Cr-sensitive PY79 strain after 4, respectively, 18 h of growth. **(B)** The accumulation of Cr by cells of Cr-tolerant NCr1a strain and Cr-sensitive PY79 strain after exposure to 1 mM Cr for 1 h with or without prior induction with a sub-inhibitory concentration of Cr(VI). “I” indicates induced sample, “UN” indicates uninduced sample. **(C)** Summary of treatment and respective average Cr content values for strains NCr1a and PY79. **(D)** Accumulation of Cr by NCr1a and PY79 cells after 18 h growth with 1 mM Cr(VI) as observed by STEM-EDS. Cr is mapped in violet color (i) High angle annular dark-field images (HAADF) of bacteria exposed to Cr together with (ii) Cr elemental distribution maps acquired by EDS, areas where the spectra were taken are marked by the yellow line.

The results show that, after exposure to 1 mM Cr(VI) for 4 h, NCr1a strain accumulates significantly less Cr than the strain PY79 of *B. subtilis*. The accumulation of Cr by NCr1a cells was consistently very low, with little variability between individual cells, specifically 0.03 ± 0.01 atomic% (Figures 1A,C). In contrast, *B. subtilis* cells showed greater variability in Cr content, making the average content of intracellular Cr significantly higher, specifically 0.27 ± 0.2 atomic% (Figures 1A,C). After 18 h of 1 mM Cr(VI) stress, the amount of Cr accumulated by NCr1a strain increased to 0.09 ± 0.03 atomic% (Figures 1A,C,D). The amount of Cr accumulated by the cells of *B. subtilis* strain after 18 h remained approximately at the same level, specifically 0.3 ± 0.23 (Figures 1A,C), suggesting that this strain could already have received a lethal dose of Cr after 4 h of exposure and prolonged incubation had no further effect. To assess whether the priming of a cell culture with sub-inhibitory concentration of Cr(VI) could induce a more effective Cr stress response, we treated the exponential-phase cultures of the strains with sub-inhibitory Cr(VI) concentration of 0.01 mM of for an additional hour. Subsequently, cells of both strains were exposed to 1 mM Cr(VI) for 1 h. Results show, that while the induction prior exposure to the high concentration had no effect on Cr accumulation by NCr1a cells (Figures 1B,C), *B. subtilis* PY79 cells clearly benefited from the pre-treatment (Figures 1B,C).

### Oxidative Stress Level in Cells of NCr1a and *Bacillus subtilis* PY79

**FIGURE 2:**
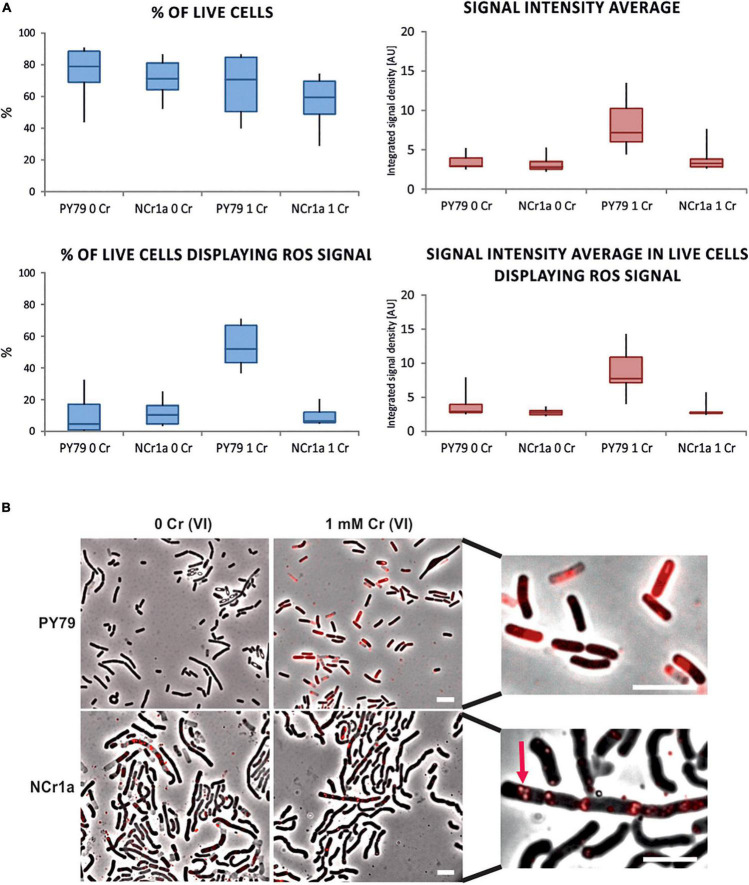
Detection of oxidative stress in cells treated with 1 mM Cr(VI) by fluorescence microscopy. CellROX™ Deep Red Reagent that emits a fluorescence signal upon oxidation was used to visualize the oxidative stress associated with Cr(VI) toxicity in cells of Cr-sensitive PY79 and Cr-tolerant NCr1a. Strains were exposed to 1 mM Cr(VI) for 6 h in minimal liquid media. **(A)** Differences in survival and signal display between samples of NCr1a and PY79 strains quantified by ImageJ software. Box plots indicate the data range for the sample of each strain grown with or without Cr(VI). At least 3,000 cells were analyzed for each sample. Statistical tests for accepting or rejecting the null hypothesis were employed and respective *p*-values were obtained. The analyzed characteristics, accurate cell counts, and *p*-values are summarized in Supplementary Table 4. **(B)** Representative fluorescence microscopy images of both strains stained by CellROX™ Deep Red Reagent: without Cr treatment (left) and after incubation with 1 mM Cr(VI) (right). Close-up images of cells stained by CellROX™ Deep Red are on the right side. Arrow marks ROS signal in presumed PHA granules in NCr1a cells. Scale bars represent 5 μm.

After Cr(VI) enters the cell, it is reduced by abundant non-specific reducing agents. The intracellular reduction of Cr(VI) is associated with turning molecular oxygen to superoxide and eventually to hydrogen peroxide or hydroxyl radicals (Shi and Dalal, 1990a,b). The degree of intracellular accumulation of Cr(VI) is thus proportional to the concentration of free radicals and the level of oxidative stress in the cell. To assess whether *B. subtilis* PY79 is subjected to greater oxidative stress than the Cr-resistant *B. pseudomycoides* NCr1a, we visualized free radicals within cells using the CellROX™ Deep Red Reagent, the fluorescent probe for detection of oxidative stress (see section “Materials and Methods”). Samples of both strains were subjected to fluorescence microscopy after 6 h of growth in the presence of 1 mM Cr(VI) and compared with the control samples grown without Cr. During this experiment, we did not see significant differences in percentage of live cells between Cr-treated or Cr-untreated samples of the same strain, or between the different strains respectively (Figure 2A), however, only translucent phase-bright cells were considered as dead (Pospíšil et al., 2020; Supplementary Table 4). One of the categories based on which we compared the strains was the CellROX™ Deep Red signal intensity (Figure 2A). The total signal intensity was calculated from the means of the values of integrated signal density recorded for each sample (see section “Materials and Methods”). This value includes the CellROX™ Deep Red signal coming from both, the live and the dead cells. In this category, the significant increase was observed only in cells of *B. subtilis* PY79 strain treated by 1 mM Cr(VI) (Figure 2A and Supplementary Table 4). The dead cells having already succumbed to Cr oxidative stress were naturally giving off substantial CellROX™ Deep Red signal. To get rid of the signal coming from already dead cells, we estimated the percentage of living cells displaying CellROX™ Deep Red signal per each sample; as well as the intensity of the CellROX Deep Red signal coming exclusively from these living cells (Figure 2A). *B. subtilis* PY79 treated by 1 mM Cr(VI) had a higher percentage of living cells displaying CellROX™ Deep Red signal than any other of the analyzed strains. In addition, also the intensity of the fluorescence signal displayed only by the living cells was the greatest in *B. subtilis* PY79 strain treated by 1 mM Cr(VI).

Interestingly, the ROS signal seemed qualitatively different in *B. subtilis* cells compared to NCr1a strain. In NCr1a cells, the CellROX™ Deep Red signals appeared to be exclusively localized in spots or patches enclosed by a membrane-like border and remained in this arrangement also in dead NCr1a cells (Figure 2B). NCr1a strain belongs to *B. cereus sensu lato* group and as such tends to form polyhydroxyalkanoate (PHA) granules during unfavorable environment conditions (Kihara et al., 2017; Ray and Kalia, 2017; Obruca et al., 2018). PHAs are highly reduced carbon-rich storage biopolymers presumably surrounded by a membrane (Obruca et al., 2018). ROS signal in NCr1a seems to localize to these intracellular structures and hints to their underestimated potential in oxidative stress alleviation.

Altogether, we have observed no significant differences between samples of NCr1a strains before or after exposure to 1 mM Cr(VI), in all the categories analyzed. On the other hand, the PY79 strain of *B. subtilis* showed major differences between treated and untreated samples in all other categories (Supplementary Table 4 and Figure 2A). Based on these results, we assume that *B. subtilis* PY79 suffers more from oxidative stress upon Cr(VI) exposure than NCr1a strain. This was in accordance with the STEM-EDS results (Figure 1C) indicating that PY79 accumulates more Cr intracellularly than the environmental *B. pseudomycoides* NCr1a strain.

### Implantation of *chrA* From *Bacillus pseudomycoides* NCr1a Improves Growth and Outgrowth of *Bacillus subtilis* PY79 Exposed to Cr(VI) Stress

Resistance to Cr can be ensured by multiple layers of protection (Nies, 2003). To assess the contribution of Cr efflux to the overall Cr resistance of *B. pseudomycoides* NCr1a strain, we were engaged in futile efforts to knock out its *chrA* locus. To be able to study the ChrA efflux from NCr1a, although in heterologous system, we cloned the *chrA* gene, together with its promoter into the *amyE* locus of the model organism, *B. subtilis* PY79. *B. subtilis* is itself moderately Cr-tolerant as it tolerates time-limited exposure to Cr(VI) in sub-millimolar concentrations and can resume the growth after the concentration of the toxic metal decreases (Garbisu et al., 1998). *B. subtilis* harbors an innate Cr efflux system in its genome, consisting of two SCHR chromate transporters *ywrB* (*chr3N*) and *ywrA* (*chr3C*), expression of which is regulated by a Cr-responsive Lrp-type regulator ChrS (Díaz-Magaña et al., 2009; Aguilar-Barajas et al., 2013). In contrast, the Cr-resistant NCr1a strain likely harbors only chromosomally encoded putative LCHR chromate transporter ChrA with no regulator sequences detected in the close vicinity of its promoter region. The putative *chrA* sequence together with its operator and promoter region was PCR extracted from the NCr1a genomic DNA and sequenced (see section “Material and Methods”). To confirm that the extracted sequence corresponds to the putative chromate transporter, the translation of this sequence was compared against the protein database as well as to the amino acid sequences of previously characterized LCHR chromate transporters (see section “Material and Methods”) from *Bacillus cereus* SJ1 (He et al., 2010); *Lysinibacillus. fusiformis* ZC-1 (He et al., 2011), *Ochrobactrum triticii* TnOtChr transposon (Branco et al., 2008), *Pseudomonas aeruginosa* pUM505 plasmid (Alvarez et al., 1999), *Shewanella oneidensis* MR-1 (Gang et al., 2019), and *Shewanella* sp. ANA-3 strain (Aguilar-Barajas et al., 2008; Supplementary Figure 1). The comparison shows the greatest homology of ChrA from NCr1a strain to a putative ChrA from *B. pseudomycoides* DSM 12442 and to the previously characterized chromate transporters of *B. cereus* SJ1 and *L. fusiformis* ZC1 with 90 and 70% of conserved residues, then to both *Shewanella* sp. with 59% of conserved residues. The lesser homology is shown to chromate tranporters from *P. aeruginosa* and *O. triticii* transposon, with conserved residues homology of 47 and 42% (Supplementary Figure 1 and Supplementary Table 5).

The putative Cr-transporter of *B. pseudomycoides* and Cr-transporter of *B. subtilis* belong to different evolutionary lines within CHR super family (Díaz-Pérez et al., 2007), which led us to question whether one could offer better protection than the other.

**FIGURE 3:**
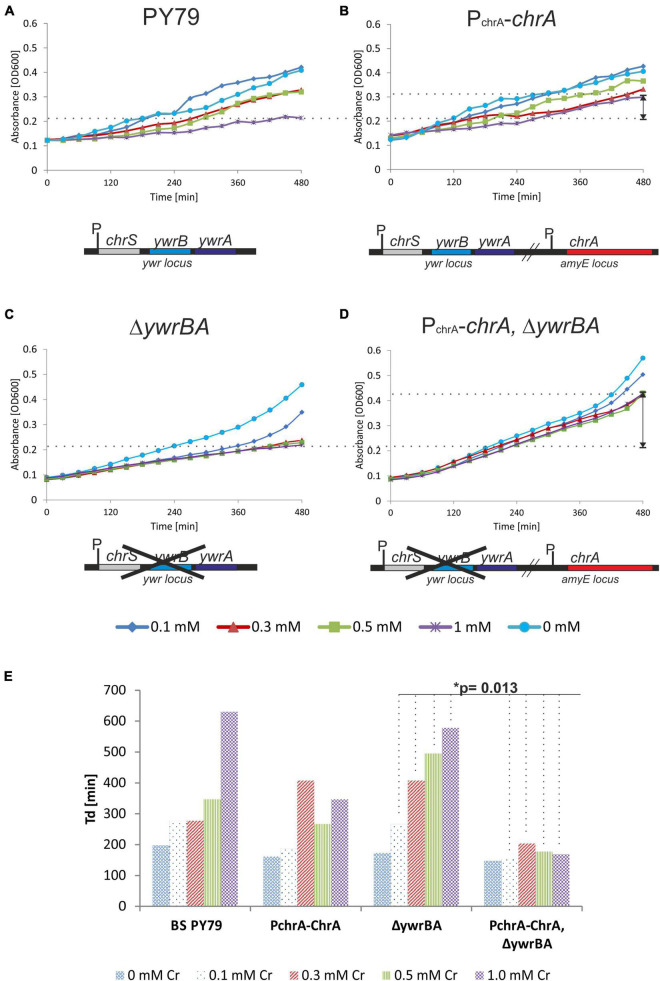
Growth of PY79 and *B. subtilis* recombinant strains affected by Cr(VI) stress. **(A–D)** The growth of strains recorded as OD600 values over time. Under each chart is a diagram of the genetic background of the strain analyzed. The legend at the bottom indicates the Cr(VI) concentration. **(A)** The growth of the reference strain PY79 under Cr stress. This strain harbors two original Cr transporters *ywrB* and *ywrA*. **(B)** The growth of the P_chrA_-*chrA* strain. This strain harbors a copy of *chrA* from NCr1a strain under control of its own P_chrA_ promoter at *amyE* locus, in addition to the original Cr transporter *ywrBA* locus of *B. subtilis.* **(C)** The growth of the Δ*ywrBA B. subtilis* strain. This strain has no Cr transporters. **(D)** The growth of the P_chrA_-*chrA* Δ*ywrBA* strain. This strain expresses *chrA* under control of P_chrA_ from *amyE* locus, in Δ*ywrBA* background. **(E)** Comparison of the doubling time (T_d_) of *B. subtilis* strains affected by different levels of Cr stress. The significance of differences was confirmed by Two-way Anova testing. The shortest T_d_ was associated with the P_chrA_-*chrA* Δ*ywrBA* genotype.

First, we studied the effect of ChrA expression on Cr tolerance of the resulting *B. subtilis* strain, referred to as the P_chrA_-*chrA* strain. To assess whether ChrA expression would compensate for the loss of YwrBA, the innate Cr efflux system of *B. subtilis* during chromate stress, we prepared the strain expressing ChrA in a Δ*ywrBA* background, referred to as strain P_chrA_-*chrA* Δ*ywrBA*. As control strains, we used a parental PY79 strain and a deletion Δ*ywrBA* strain. These strains were subjected to chromate stress represented by varying concentrations of Cr(VI) in the liquid culture. The growth of each strain was recorded as OD600 values over time and was taken as an indicator of tolerance for, or susceptibility to Cr(VI). A mild increase of *B. subtilis* Cr(VI) tolerance in the presence of 1 mM Cr(VI) was observed when ChrA was expressed in the wild-type background (Figures 3A,B). The Δ*ywrBA* strain displays Cr sensitivity, albeit it still exhibits some growth in the presence of 0.1 mM Cr(VI) (Figure 3C). The inability of Δ*ywrBA* to adapt to other sub-millimolar concentrations of Cr(VI) indicates that chromate efflux transporters might be the key determinants of survival under sub-millimolar Cr(VI) stress for *B. subtilis* (Figures 3A,C). On the other hand, wildtype PY79 and the Δ*ywrBA* strain display similar growth in the presence of 1.0 mM Cr(VI), suggesting that, for PY79 wild-type strain, there is no effective mechanism to fend off Cr stress at this Cr(VI) concentration. Expression of ChrA in the Δ*ywrBA* background results in elevated Cr tolerance across the spectrum of all tested Cr(VI) concentrations, especially at 1 mM Cr(VI) (Figures 3B–D). Hence, ChrA from the environmental *B. pseudomycoides* strain NCr1a is not only able to compensate for the absence of the chromate transporter operon *ywrBA*, but seems to increase Cr tolerance at concentrations of Cr(VI) otherwise inhibitory to the parental PY79 strain (Figures 3A,D). The doubling time (Td) was estimated from the exponential phase of growth of each strain for each level of Cr stress tested (see section “Materials and Methods”) (Figure 3E and Supplementary Figure 2). The shortest doubling time across all concentrations of Cr(VI) is associated with the P_chrA_-*chrA* Δ*ywrBA* strain, whereas the doubling time of P_chrA_-*chrA* strain is shorter at 1 mM Cr(VI) than that of PY79 strain (Figure 3E and Supplementary Table 6). The differences between doubling times of strains were found to be significant by statistical testing using Two-way Anova without replication (Supplementary Table 7). From all strains tested only P_chrA_-*chrA* Δ*ywrBA* displays statistically significant differences in T_d_ across the concentrations of Cr(VI) used when compared to the parental Δ*ywrBA* strains as well as PY79 strain (Supplementary Table 8). This approach, however, only tells how fast the mass is doubled in the fastest phase of growth, and doesn’t consider other phases, such as the length of the lag phase, the stationary phase, or the onset of decline.

It is well established that Cr stress leads to damage of DNA, lipids and proteins (Roundhill and Koch, 2002), thus the defects in cell division in susceptible strains are inevitable. The method based on measuring OD600 over time, however, partially covers up these defects since both, accumulation of debris as well as elongation of cells (instead of division) add up to overall optical density of the culture.

**FIGURE 4:**
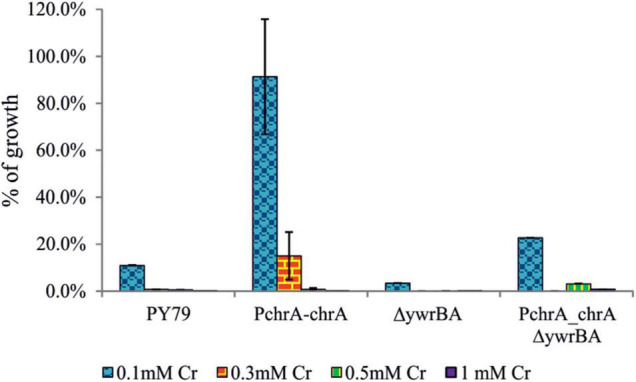
The CFU outgrowth of strains after exposure to different levels of Cr stress. After 6 h of growth in M9 supplemented with Cr(VI), cultures were serially diluted and plated onto M9 agar media. CFU counts of strains affected by Cr stress were expressed as a percentage of CFU count of strains grown without Cr(VI). Different levels of Cr stress were represented by different concentrations of Cr(VI) in media. For each strain and Cr(VI) concentration, the experiment was performed at least in triplicate. In some cases, the standard error bars are too small to be visible.

Thus we tested the colony outgrowth of strains subjected to different levels of Cr(VI) stress, to discern the successful division events. The method used is based on the count of viable colonies formed after cultures were exposed to Cr(VI) for 6 h, serially diluted and plated onto minimal agar media without Cr (see section “Materials and Methods”). Strains grown without Cr(VI) and treated afterward in the same manner, served as a reference. The ability of cells to successfully divide and their progeny to grow is referred to as the “fitness” of strains in this study and was expressed as the percent CFU to that of the reference strain (Figure 4). Results show that even the lowest concentration of Cr(VI) (0.1 mM) affects the ability of PY79 strain to divide and grow. With increasing concentrations of Cr(VI), the number of colonies decreases dramatically, which is likely attributable to intracellular accumulation of Cr(III) and/or overall damage linked to oxidative stress. On the other hand, we see that strains expressing ChrA fare better when exposed to 0.1 mM Cr(VI) than parental strains PY79 and Δ*ywrBA*. These results suggest that the expression of *chrA* under the control of its native promoter increases Cr tolerance in cells exposed to sub-inhibitory concentrations of Cr at least during the first 6 h from the start of exposure (Figure 4). After exposure of strains to the higher Cr(VI) concentration (0.3 mM), the ChrA expression in P_chrA_-*chrA* strain improves outgrowth of colonies. However, this is not the case for the P_chrA–_*chrA*Δ*ywrBA* strain, which displays zero outgrowth at this level of Cr stress but shows some outgrowth at 0.5 mM (Figure 4).

Overall, during both the growth and the outgrowth experiments we observed the positive effect of ChrA expression on the survival of *B. subtilis* strains exposed to Cr(VI) stress.

### Cell Morphology and Division Defects After Exposure to Cr(VI)

**FIGURE 5:**
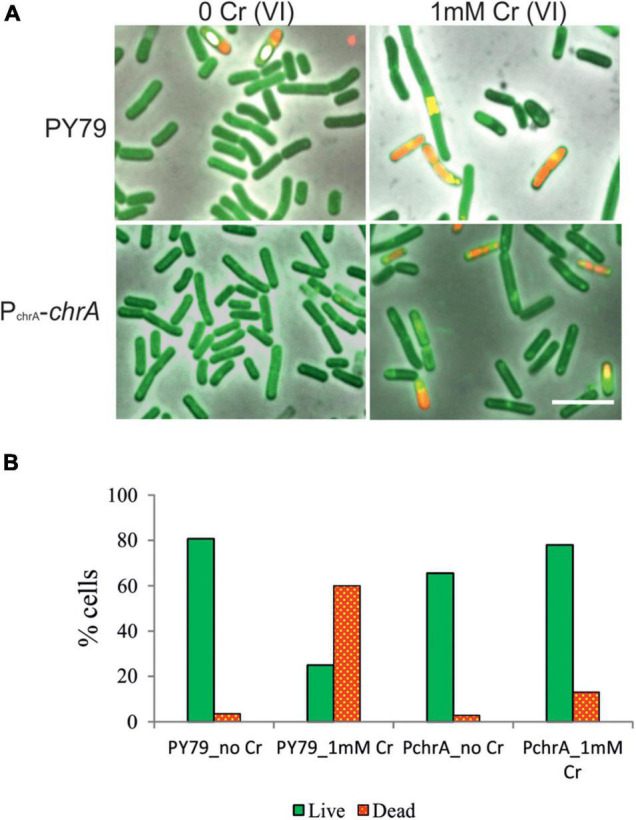
Live/Dead staining of *B. subtili*s PY79 and P_chrA_-ChrA strain. **(A)** Fluorescence microscopy images of Live/Dead staining of PY79 and P_chrA_-ChrA strains exposed to 1 mM Cr(VI) for 6 h, together with respective controls grown without Cr. After exposure to Cr(VI) cells of both strains tend to be longer, with multiple shape irregularities. The panels are overlay of phase contrast and fluorescence images. The scale bars represent 5 μm. **(B)** Statistics of the Live/Dead staining experiment. The number of live cells (green, stained by Rhodamin 123) and the number of dead cells (red, stained by PI) were expressed as a percentage of all cells in recorded images. At least 750 cells were analyzed for each sample.

To visualize the effect of Cr(VI) on vital processes and at the same time assess cell survival, we employed Live/Dead fluorescence staining of bacterial cultures exposed to Cr stress (see section “Materials and Methods”) and fluorescence microscopy analysis. This approach provides us with direct insight into the cell-specific response to toxic stress, focusing especially on morphological changes and the effect of ChrA expression on the Live/Dead cells ratio. The principle of the staining is the diverse affinity of fluorescent stains to lipid membranes. Lipophilic Rhodamin 123 stains cells with intact membranes, but water-soluble Propidium Iodide is able to enter cells only when the cytoplasmic membrane is breached (Jepras et al., 1995). We compared samples of the parental PY79 and the P_chrA_-*chrA* strains. Since the differences were the most obvious after exposure to extreme Cr stress, the strains were cultivated in media with 1 mM Cr(VI) for 6 h. As a result of Cr stress, both strains showed elongated and otherwise misshapen cells, indicating impairment of cell division (Figure 5A). Live/Dead staining revealed that even some phase-dark cells were stained by PI, indicating that they were dead although appeared alive. According to the statistics from Live/Dead fluorescence microscopy imaging, the parental PY79 strain displays more than 50% dead cells after cultivation with 1 mM Cr(VI). On the other hand, P_chrA_-*chrA* strain shows only 13% of cells as dead, after the same treatment (Figure 5B).

### Expression of GFP-ChrA in *Bacillus subtilis* Cells

**FIGURE 6:**
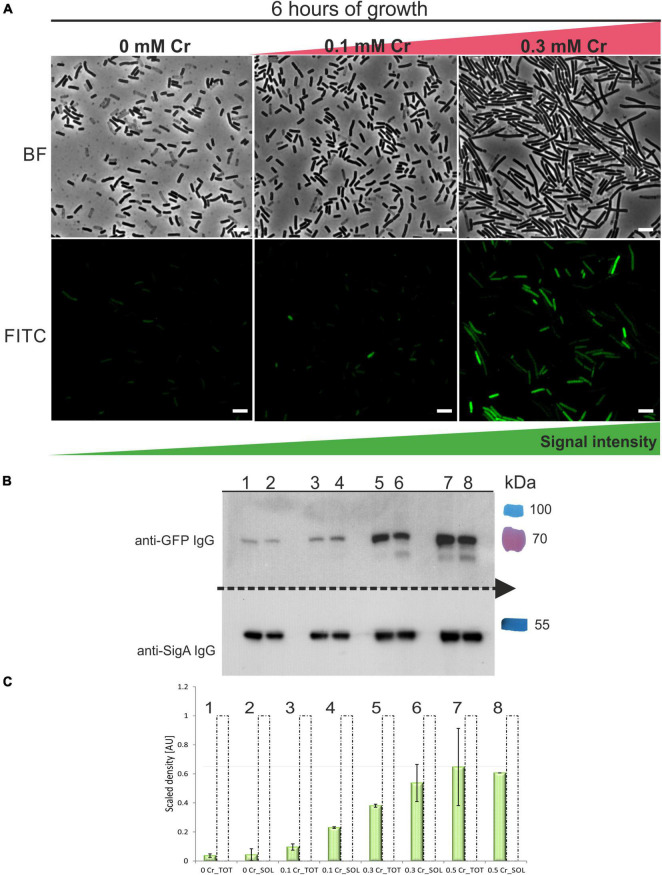
Expression of GFP-ChrA in *B. subtilis* strain exposed to different Cr(VI) stress. **(A)** Fluorescence microscopy of GFP-ChrA in cells of *B. subtilis* subjected to Cr stress. Upper panel shows bright-field images, bottom panel shows fluorescence images of the strain P_chrA_-*gfp-chrA*. GFP-ChrA fusion is expressed under the control of its *chrA* promoter in *B. subtilis* PY79. With increasing concentrations of Cr(VI), the number of cells expressing GFP-ChrA increases as does the length of cells and the intensity of the GFP signal. The scale bars represent 5 μm. **(B)** Western blot analysis of P_chrA_-*gfp-chrA* lysates using anti-GFP and anti-Sig70 antibodies. The Western blot membrane was cut in half. Each half was incubated with the appropriate IgG to be able to detect both GFP-ChrA and SigA simultaneously, for each sample. Lanes 1–2 represent samples incubated without Cr, lanes 3–4 are samples incubated with 0.1 mM Cr(VI), lanes 5–6 are samples incubated with 0.3 mM Cr(VI) and lanes 7–8 are samples incubated with 0.5 mM Cr(VI). The last color lane represents the protein molecular weight standards in kDa (PageRuler™ Plus Prestained). **(C)** Quantification of the GFP-ChrA signal in total and soluble fractions of samples subjected to varying Cr stress. The signals were quantified by the ImageJ software. The data are labeled by numbers of the corresponding lanes on the Western blot. Dashed unfilled columns represent the relative amount of SigA normalized to 1. Green-filled columns represent the relative amount of GFP-ChrA normalized to the amount of SigA.

To assess whether ChrA is stably expressed in *B. subtilis* PY79 and to monitor its expression during increasing Cr(VI) concentrations, we prepared N-terminal in-frame fusion of *mgfp* to the *chrA* coding sequence. The fusion was placed under the control of *chrA* upstream region containing the putative P_chrA_ promoter and introduced into the *B. subtilis amyE* locus. The expression of the GFP-ChrA in the resulting P_chrA_-GFP-ChrA strain was monitored using fluorescence microscopy and anti-GFP IgG Western blotting (see section “Materials and Methods”). GFP fluorescence microscopy revealed that with increasing concentration of Cr(VI) the length of the cells also increases, indicating the inhibition of cell division. More importantly, the intensity of the GFP signal grows with the increasing concentration of Cr(VI) as well (Figure 5A). To be able to quantify the amount of GFP-ChrA expressed for each concentration of Cr(VI), P_chrA_-GFP-ChrA lysates were subjected to anti-GFP Western blotting. To obtain a reference standard for each sample, the concentration of which does not vary with changing conditions, we chose to simultaneously visualize the housekeeping protein SigA of *B. subtilis* using anti-Sig70 antibody (see section “Materials and Methods”). This set-up allowed quantification of the relative amounts of SigA in each sample and normalization to the amount of GFP-ChrA for each sample. The ImageJ software was used for quantification of protein bands (see section “Material and Methods”). Western blot analysis of P_chrA_-GFP-ChrA using anti-Sig70/anti-GFP IgG suggests that there is only a minimal amount of GFP-ChrA expressed in cells grown in the absence of Cr (Figures 6B,C). For concentrations of Cr(VI) from 0 to 0.3 mM it seems that with increasing concentration of Cr(VI), the GFP signal is also intensified (Figures 6A–C), indicating the inducibility of the P_chrA_ promoter by the presence of Cr(VI). Considering that ChrA from *B. pseudomycoides* and YwrBA from *B. subtilis* are chromate transporters belonging to different families, the mechanism of Cr efflux activation by the presence of Cr itself is likely conserved in these species. According to the GFP signal, however, the concentration of the fusion protein in cells doesn’t seem to increase any further at 0.5 mM Cr(VI). Either the expression of the fusion is stalled or even declines or the protein starts to be degraded at this concentration of Cr(VI).

## Discussion

This study investigates the underlying process resulting in the remarkable resistance to Cr(VI) exhibited by the environmental strain NCr1a, isolated from a pristine soil and identified in our previous study as *B. pseudomycoides* (Tamindžija et al., 2019). Interestingly, this strain was able to tolerate an 8 mM concentration of K_2_CrO_4_, or Cr(VI), in minimal media, while the laboratory reference strain *B. subtilis* PY79 was able to withstand only a maximum of 0.5 mM Cr(VI). Both strains are originally isolated from a niche in which they could not experience exposure to Cr; however, NCr1a is able to tolerate high levels of Cr(VI) stress. The study has also shown that the *B. subtilis* PY79 strain has similar Cr(VI) reduction abilities to NCr1a strain; the only difference was that it did not tolerate high Cr(VI) stress (Tamindžija et al., 2019). Here, we probe the important question of the mechanism ensuring survival in the presence of Cr(VI). STEM-EDS microscopy enabled us to detect Cr accumulated by individual cells of both abovementioned strains. From that analysis, it was apparent that under Cr(VI) stress, the cells of NCr1a accumulated less Cr than PY79 cells. While there was almost no variation in Cr content amongst NCr1a cells, these variations were large amongst PY79 cells. Bacteria employ multiple mechanisms to prevent the entry of Cr(VI) into the cell, Cr efflux being one of them. Our experimental hypothesis was that the Cr efflux pump of the NCr1a strain, ChrA, may be more effective than its PY79 counterpart, YwrBA. This seemed to be supported by the fact that the two efflux pumps belong to divergent families within the CHR superfamily, that their genetic determinants are differently organized in the genome, and their expression is dissimilarly regulated (Díaz-Pérez et al., 2007; Aguilar-Barajas et al., 2013).

As the genetic manipulation of NCr1a strain has been unsuccessful so far, our experiments were focused on its putative Cr transporter ChrA operating in the heterologous *B. subtilis* PY79 system. We prepared recombinant strains of *B. subtilis* carrying a copy of *chrA* under control of its promoter, either in the wild type or in Δ*ywrBA* background. In the first set of experiments, we compared the growth of the wildtype and recombinant strains under different levels of Cr stress. We wondered whether the accumulation of multiple Cr transporters in *B. subtilis* results in a more robust extrusion of Cr(VI), as is seen in some other species (Reyes-Gallegos et al., 2016). Another issue we addressed was whether ChrA, an LCHR chromate transporter, compensates for the loss of SCHR transporters YwrB and YwrA in the Δ*ywrBA B. subtilis* strain. The expression of ChrA in the wildtype background increased the Cr tolerance only in the presence of 1 mM Cr(VI). On the other hand, the growth of Δ*ywrBA* strain compared to that of the wild type PY79 at 1 mM Cr(VI) suggests that YwrBA efflux is not operational at this level of Cr stress in *B. subtilis* wild type strain. The *ywrBA* genes are organized in an operon, regulated by the ChrS regulator (Aguilar-Barajas et al., 2013). It is hypothesized that this regulator hinders the expression of either of the proteins in case of limited uptake of sulfur-containing nutrients (Ramírez-Díaz et al., 2008; Aguilar-Barajas et al., 2013). The regulation of YwrBA thus prevents co-extrusion of essential sulfate together with the toxic chromate. It is therefore possible, that in minimal media, after several hours the sulfate supply decreases, and the efflux pump is shut to prevent loss of the nutrient. In the *B. pseudomycoides* genome, no analogous regulator of Cr efflux was found. After implantation of *chrA* into the *B. subtilis* PY79, we observed the increase of Cr tolerance in liquid medium containing 1 mM Cr(VI), as well as the increase of Cr-tolerance across almost all tested Cr(VI) concentrations in the case of P_chrA_-*chrA*Δ*ywrBA* strain. Our results indicate that expression of ChrA not only rescues the Cr sensitive phenotype of Δ*ywrBA* strain but elevates the Cr-tolerance of P_chrA_-*chrA*Δ*ywrBA* strain to levels above that of the wild type. Experiments focused on the survival of Cr-affected strains after plating showed that expression of ChrA also improves CFU outgrowth either in the wild type or Δ*ywrBA* background. However, there are discrepancies in the outcomes of the metal susceptibility methods used, as the colony outgrowth experiment showed much lower Cr tolerance in engineered strains when compared to OD600 method. In addition, the matter of the potential benefit of accumulation of chromate transporters in the genome of *B. subtilis* cannot be answered unambiguously using these methods. While OD600 approach suggests that the presence of YwrBA and its regulator interferes with ChrA activity (or vice versa), the CFU approach shows better survival of the strain expressing both types of efflux pumps under mild Cr stress. It has been reported that under unfavorable conditions, the immobilization of bacteria tends to limit their growth in comparison with planktonic growth due to worse access to nutrients in solid media (Jeanson et al., 2015), which might be in theory an explanatory hypothesis for our observation. A strain with unhinged expression of efflux pump, P_chrA_-*chrA* Δ*ywrBA*, probably lost more sulfate, which would be difficult to replenish after transition to minimal solid media. On the other hand, a strain with two types of efflux pumps, however, possibly interfering with each other would have a greater reserve of sulfate and would fare better on the minimal solid media, after experiencing only a mild Cr stress. Overall, our results show that implantation of *chrA* into *B. subtilis* PY79 increased its Cr-tolerance; however, this was far below the level observed for environmental strain NCr1a strain (Tamindžija et al., 2019). This indicates that very likely the NCr1a strain of *B. pseudomycoides* has additional layers of protection against Cr stress and that its Cr efflux is only moderately more effective than that of *B. subtilis* PY79.

The expression of GFP-ChrA in *B. subtilis* PY79 reveals that it could be induced by intracellular Cr only. We are wildly speculating that this might be mediated through a conserved metal-sensor protein (Ma et al., 2009; Waldron et al., 2009; Chandrangsu et al., 2017). The mechanism of Cr efflux activation might be ancient, given the fact that the P_chrA_ promoter is recognized and *chrA* expression was activated in the heterologous host of *B. subtilis* PY79. Notably, not every cell seems to express the GFP-ChrA fusion protein, indicating the population heterogeneity in response to environmental stress (Fernandes et al., 2011). This finding agrees with the results of STEM-EDS microscopy, where significant variations in Cr accumulation were observed in the case of the *B. subtilis* PY79 strain. In *B. subtilis*, the expression of YwrBA seems to be tightly regulated (Aguilar-Barajas et al., 2013). Our bioinformatic analysis did not reveal the presence of any potential negative regulator upstream of *chrA* efflux gene in *B. pseudomycoides*. The possible absence of regulation components of Cr efflux also hints at the existence of other mechanisms to eliminate the intracellular Cr stress in *B. pseudomycoides*. Experiments aimed at the detection of oxidative stress in both strains revealed that under Cr stress, the NCr1a strain likely accumulates free radicals in vesicles known as PHA granules as it was shown for strains of *B. cereus sensu lato* group (Kihara et al., 2017; Obruca et al., 2018). These granules are packed with a reduced biopolymer, able to sequestrate free radicals, and are known to have a key role in eliminating the oxidative stress caused by a cold shock (Ayub et al., 2009). PHA granules might thus be a good candidate for playing a leading role in mitigating intracellular Cr(VI) stress in the NCr1a strain. Conversely, in the case of *B. subtilis* PY79, the YwrBA efflux pump seems to be the main determinant of its survival strategy during Cr stress. Indeed, deletion of the *ywr* operon resulted in a strain that struggled with the sub-millimolar concentrations of Cr(VI). *B. subtilis* PY79 does not produce PHA, on the other hand, its YwrBA efflux pump is so thoroughly regulated that its performance does not interfere with the ability of the cell to fight oxidative stress, since that is mediated through sulfur-containing reducing compounds and enzymes (Holland and Avery, 2011). The downside of such Cr efflux regulation might be that it offers protection only at sub-millimolar concentrations of Cr(VI). *B. subtilis* wields also other mechanisms to fend of Cr stress, such as NfrA, a member of nitro/flavinreductase family (Zheng et al., 2015), homolog of NfsA from *E. coli*. In *B. subtilis*, this effective universal reductase has a broad electron acceptor activity and it can reduce substrates using NAD(P)H as an electron donor (Zenno et al., 1998).

Overall, our results suggest that the Cr efflux system contributes considerably to the Cr tolerance of *Bacillus* spp. Under Cr stress, the survival strategy of *B. subtilis* PY79 seems to be different from that of the NCr1a strain. Although the NCr1a strain harbors an effective chromate efflux system, it seems to be equipped with several other mechanisms which safeguard its vegetative growth even during highly elevated Cr(VI) concentrations at nutrient-limited conditions. Despite being an efficient tool for the elimination of Cr stress, the *B. subtilis* YwrBA efflux system seems to offer only limited protection, insufficient to sustain its vegetative growth in such conditions. Since the *B. subtilis* strain PY79 we used in this study is a laboratory reference strain, it cannot be excluded that environmental isolates of *B. subtilis* possess additional survival mechanisms more effective to fight off Cr stress.

## Data Availability Statement

The original contributions presented in the study are included in the article/Supplementary Material, further inquiries can be directed to the corresponding author/s.

## Author Contributions

ZC: conception of the study, experiments and their design and manuscript writing. RC: experiments and manuscript edition. DT: experiments, mainly STEM-EDS techniques and manuscript edition. DR: funding acquisition and manuscript edition. RB-L and IB: conception of the study, funding acquisition and manuscript edition. All authors contributed to the article and approved the submitted version.

## Conflict of Interest

The authors declare that the research was conducted in the absence of any commercial or financial relationships that could be construed as a potential conflict of interest.

## Publisher’s Note

All claims expressed in this article are solely those of the authors and do not necessarily represent those of their affiliated organizations, or those of the publisher, the editors and the reviewers. Any product that may be evaluated in this article, or claim that may be made by its manufacturer, is not guaranteed or endorsed by the publisher.

## Footnotes

1
https://lukemiller.org/index.php/2010/11/analyzing-gels-and-western-blots-with-image-j/

2
https://imagej.nih.gov/
