# Supplementary material for: Implantation of Bacillus pseudomycoides Chromate Transporter Increases Chromate Tolerance in Bacillus subtilis
Source: Front Microbiol. 2022 Mar 7;13:842623. doi: 10.3389/fmicb.2022.842623 (PMC8940164; doi:10.3389/fmicb.2022.842623)
Supplement: Supplementary file 1 [file Data_Sheet_1.docx]

Supplementary material

**Table S1 Primers used**

| **Primer** | **Sequence (5’3’)** |
| --- | --- |
| chrABP_Up_AccF | GATGATGATGGTACCCAGCTTAGCAACAAAATGTTTTGTCATACTAG |
| chrABP_STOP_BamR | GATGATGATGGATCCCTAGATAATAGAAAGAATATAACCGCTTAAAGCAC |
| U1000_ywrB_BamF6 | GATGATGATGGATCCCCTGAACGGCCGCTGC |
| U1000_ywrB_SalR2 | GATGATGATGTCGACCTCTCCTTTACAGCTTCATTCCCATAC |
| D1000_ywrA_SalF2 | GATGATGATGTCGACCCAGAAATGCCGTTTGATATGACAG |
| D1000_ywrA_EcoRIR | GATGATGATGAATTCCATAATTAAGCATTTTGGCGGTTAG |
| Kan_new_SalF | GATGATGATGTCGACGTGGTTTCAAAATCGGCTCCGTC |
| Kan_new_SalR2 | GATGATGATGTCGACCATCAGAGTATGGACAGTTGCGGATGTAC |
| Up_chrABP_Prom_AccF | ATCATCATCGGTACCCAGCTTAGCAACAAAATGTTTTGTCATACTAG |
| Up_chrABP_Prom_AccR | ATCATCATCGGTACCGTCCTCCAAAAGAAGTAAGGCCTAAC |
| chrABP_GFP_EcoF | GATGATGATGAATTCGTGGCTCATCTTGGGTACTTTCATCAC |
| chrABP_GFP_EcoR | GATGATGATGAATTCCTAGATAATAGAAAGAATATAACCGCTTAAAGCAC |

**Table S2 Plasmids used**

| **Plasmid** | **Origin** |
| --- | --- |
| pSG1729 | (Feucht and Lewis, 2001) |
| pSG17P_chrA_-*chrA* | This study |
| pSG17P_chrA_-*gfp*-*chrA* | This study |
| pMAD | (Arnaud *et al.*, 2004) |
| pMAD Up_*ywrB*:*kan*:down_*ywrA* | This study |

**Table S3 Bacterial strains used**

| **Strain** | **Origin** |
| --- | --- |
| *E.coli* MM294 | (Backman *et al.*, 1976) |
| *E.coli* DH5α | (Invitrogen) |
| *B. pseudomycoides* NCr1a | (Tamindžija *et al.*, 2019) |
| *B. subtilis* PY79 | (Youngman *et al*., 1984) |
| *B. subtilis* P_chrA_*-chrA* | This study |
| *B. subtilis* Δ*ywrBA* | This study |
| *B. subtilis* P_chrA_*-chrA* Δ*ywrBA* | This study |
| *B. subtilis* P_chrA_-*gfp*-*chrA (amyE::_PchrA_-gfp-chrA spc)* | This study |

**Supporting file S1 Statistical analysis of the sample differences**

*Box plots*

For visual manifestation of differences between samples, the box plots were created. For creating a box plot graph, the Min, and Max values for each dataset were estimated, as well as the first, the second and the third quartile (Q1, Q2, Q3). Whiskers of the box plot represent extreme values within dataset, while the body of the box plot expresses the difference between the Q3 and the Q2 (upper box), the median or Q2 (the line in between boxes) and the difference between the Q2 and the Q1 (lower box). The Q1 plot remains hidden and designates the position of the particular box plot on y axis.

*Statistical evaluation of differences in ROS signal display*

All statistical analysis was performed in MS Excel. After collection of data using ImageJ v1.53f, the integrated density data sets of each image were averaged; the total number of cells recorded and the percentage of live cells, live cells displaying ROS and live cells displaying the over-average ROS signal assessed. For each sample, there were 6 fields/images analysed, with at least 3000 cells in total. The values characteristic for each sample were finalized by addition of cell counts from all images belonging to one particular sample and averaging the datasets of integrated density between them and estimating the standard errors. To assess the existence of statistically significant differences between Cr-treated and Cr-untreated sample of one strain, the datasets originating from the analysis of the images representing each sample were subjected to two-sample t-testing assuming equal variances. To assess the existence of statistically significant differences between equally treated samples of two different strains, the datasets were first subjected to Levene’s test for equality of variances, during which absolute residuals were calculated firstly, using the ABS function and the MEAN for each value of the dataset. Then single factor ANOVA function was used to assess variance between datasets, with H0 assuming variances between the datasets are not equal. After obtaining p-value 37%, with alpha value set to 5%, the H0 was accepted. To test the actual significance of differences, the datasets were then subjected to two-sample t-testing assuming unequal variances. The null hypothesis in case of both types of t-testing was that there are no significant differences and the alpha value was set to 5%. Thus p-values lower than alpha implied the H0 should be rejected, while p-values higher than alpha implied H0 should be accepted.

**Table S4 Statistical assessment of differences in oxidative damage display between strains treated by Cr(VI)**

**A**

| **PY79** | **CELL COUNT** | | | **SIGNAL INTENSITY [AU]** | | |
| --- | --- | --- | --- | --- | --- | --- |
|  | TOTAL | % LIVE | % LIVE ROS | AVERAGE | AVERAGE IN LIVE ROS | AREA [pix˄2] |
| **0 mM Cr** | 3524 | 78.9 | 7.9 | 3.46 +/- 1.02 | 3.87 +/- 2.05 | 7.59 |
| **1 mM Cr** | 3064 | 68.3 | 51.8 | 8.19 +/- 3.4 | 8.77 +/- 3.66 | 80.04 |
| **P(T<=t) one-tail [%]** | | 24.2 | 0.014 | 0.45 | 0.84 | 0.028 |
| **P(T<=t) two-tail [%]** | | 48.4 | 0.027 | 0.9 | 1.69 | 0.056 |
| **Differences** | | N | S | S | S | S |

**B**

| **NCr1a** | **CELL COUNT** | | | **SIGNAL INTENSITY [AU]** | | |
| --- | --- | --- | --- | --- | --- | --- |
|  | TOTAL | % LIVE | % LIVE ROS | AVERAGE | AVERAGE IN LIVE ROS | AREA [pix˄2] |
| **0 mM Cr** | 4823 | 72.1 | 10.9 | 3.22 +/- 1.1 | 2.81 +/- 0.49 | 11.13 |
| **1 mM Cr** | 4601 | 61.4 | 10.1 | 3.92 +/- 1.86 | 3.17 +/- 1.22 | 17.35 |
| **P(T<=t) one-tail [%]** | | 6.4 | 31.1 | 22.4 | 25.8 | 13.7 |
| **P(T<=t) two-tail [%]** | | 12.8 | 62.2 | 44.9 | 51.5 | 27.3 |
| **Differences** | | N | N | N | N | N |

**C**

| **0 mM Cr** | **CELL COUNT** | | | **SIGNAL INTENSITY [AU]** | | |
| --- | --- | --- | --- | --- | --- | --- |
|  | TOTAL | % LIVE | % LIVE ROS | AVERAGE | AVERAGE IN LIVE ROS | AREA [pix˄2] |
| **PY79** | 3524 | 78.9 | 7.9 | 3.46 +/- 1.02 | 3.87 +/- 2.05 | 7.59 |
| **NCr1a** | 4823 | 72.1 | 10.9 | 3.22 +/- 1.1 | 2.81 +/- 0.49 | 11.13 |
| **P(T<=t) one-tail [%]** | | 34.5 | 42.3 | 35.2 | 13.3 | 22.9 |
| **P(T<=t) two-tail [%]** | | 69.1 | 84.6 | 70.3 | 26.7 | 45.8 |
| **Differences** | | N | N | N | N | N |

**D**

| **1 mM Cr** | **CELL COUNT** | | | **SIGNAL INTENSITY [AU]** | | |
| --- | --- | --- | --- | --- | --- | --- |
|  | TOTAL | % LIVE | % LIVE ROS | AVERAGE | AVERAGE IN LIVE ROS | AREA [pix˄2] |
| **PY79** | 3064 | 68.3 | 51.8 | 8.19 +/- 3.4 | 8.77 +/- 3.66 | 80.04 |
| **NCr1a** | 4601 | 61.4 | 10.1 | 3.92 +/- 1.86 | 3.17 +/- 1.22 | 17.35 |
| **P(T<=t) one-tail [%]** | | 18.9 | 0.01 | 1.4 | 0.6 | 0.2 |
| **P(T<=t) two-tail [%]** | | 37.9 | 0.02 | 2.8 | 1.2 | 0.5 |
| **Differences** | | N | S | S | S | S |

The statistical assessment of differences between samples of PY79 and NCr1a strains without or with addition of Cr(VI). Arrays of data sets characterizing samples in ROS signal distribution and intensity were subjected to statistical analysis encompassing Levene’s test for equality of variances and subsequent T-testing of the differences. Given the null hypothesis assumes that there are no differences between analysed samples, P-values at the bottom of each column represent the likelihood of the experiment outcome to happen by chance. The probability level was set to 5%, hence P-values lower than this indicates that the null hypothesis can be rejected and there are significant differences between samples. P-value higher than 5% means that the null hypothesis failed to be rejected and that the differences between samples are not statistically significant. In the bottom rows of each table, P-values expressed in percentage are shown. P-values lower than 5% indicate the statistical significance of differences, marked as “S”. P-values higher than 5% indicate that the differences are not statistically significant; this case is marked as “N”. A- B.) The differences between differently treated samples of the same strain were analysed using t-test assuming equal variances. Cr stress induced differences in percentage of live cells giving off ROS signal as well as in signal intensity are significant only in PY79 strain. C-D.) The differences between samples of different strains subjected to the same treatment (either 0 mM Cr in media or 1mM Cr in media) were analysed using t-test assuming unequal variances. While the strains NCr1a and PY79 are not subjected to Cr stress, no significant differences in percentage of cells giving off ROS signal or in signal intensity can be seen. After these strains are exposed to Cr stress, the differences are significant in all characteristics related to ROS signal.

A


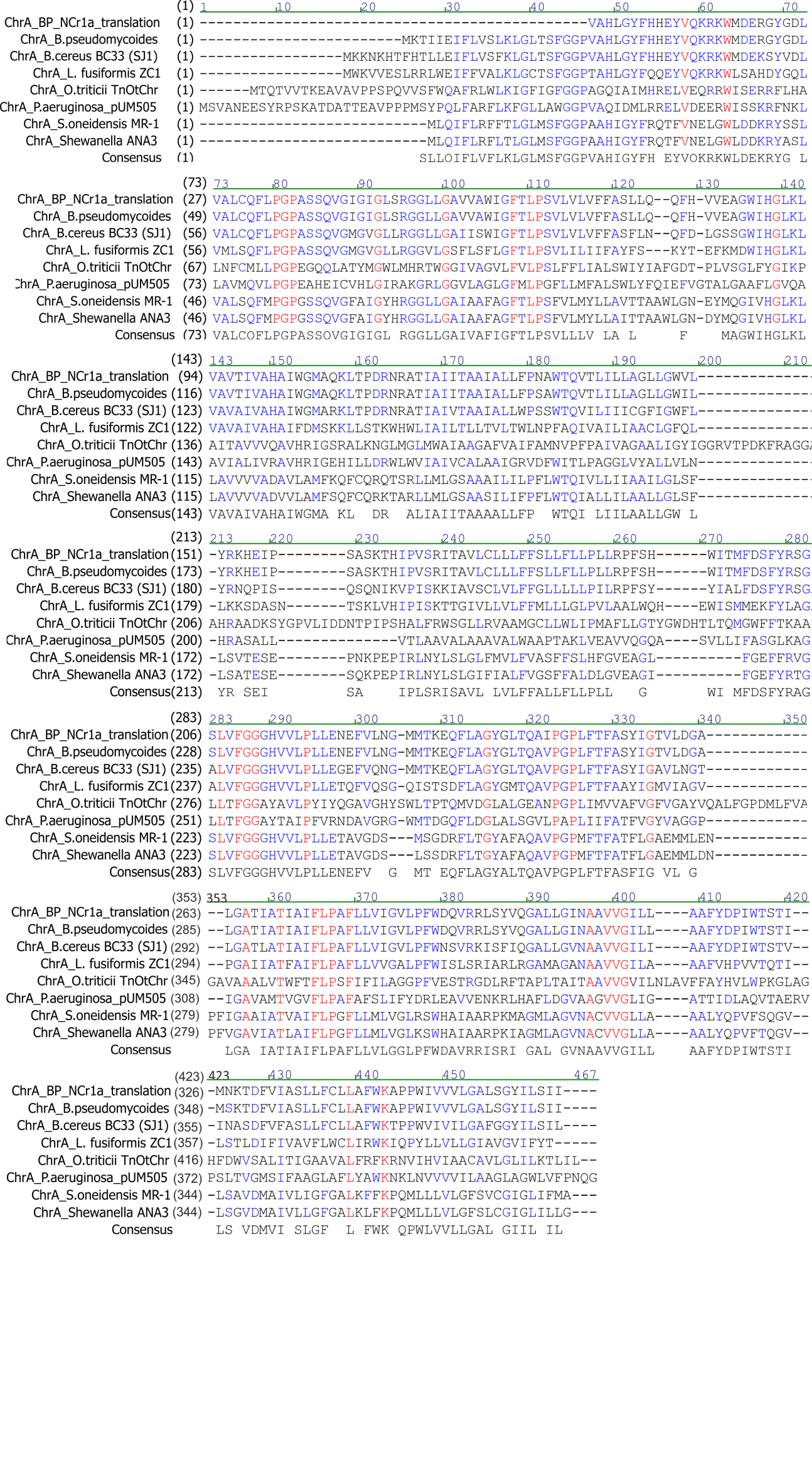


B

CAGCTTAGCAACAAAATGTTTTGTCATACTAGCAACATTCTCCTTATATAAATTTCAATTTGAATAGCTGGTATCTCTTTAACTAATTTATTGCCGGGACACGATTAATTATGACTCTTTCAGAAATGCTTTTTCATGATGGTTATTCTACTCAAGAAATATTTACAAAAATATTACATAACAATCATTCCTGACAGCACACTGCTTACTATATAATGCTCGTAGATTTAAAATTTCTTTAGGAGGTTCTTCTTGAAAACAATAATAGAAATCTTTCTCGTATCACTCAAGTTAGGCCTTACTTCTTTTGGAGGACCCGTGGCTCATCTTGGGTACTTTCATCACGAATATGTACAAAAACGAAAATGGATGGATGAGCGGGGCTATGGGGATTTAGTGGCACTCTGTCAATTTCTACCCGGTCCTGCAAGCAGCCAAGTTGGAATTGGCATCGGTTTATCACGAGGCGGGCTCCTTGGAGCTGTAGTCGCTTGGATTGGCTTTACACTCCCGTCAGTTCTCGTTCTTGTTTTTTTCGCTTCACTACTCCAGCAATTTCATGTGGTGGAAGCTGGCTGGATTCATGGATTAAAACTTGTAGCAGTAACGATTGTCGCACATGCAATATGGGGAATGGCACAGAAATTAACACCGGACCGTAACCGAGCAACAATTGCAATTATTACTGCTGCAATTGCGTTATTATTTCCAAACGCTTGGACACAAGTTACGCTTATTCTACTTGCTGGTCTTCTCGGTTGGGTCTTATATCGTAAGCATGAAATCCCATCTGCTAGCAAAACACATATTCCTGTTTCACGTATAACAGCTGTCCTTTGTCTCTTACTATTCTTTAGCTTATTATTCTTGTTACCACTATTACGACCATTTTCGCACTGGATTACCATGTTTGATAGTTTCTATCGCTCTGGCTCACTCGTGTTTGGCGGAGGACATGTCGTTCTTCCCCTTCTTGAAAATGAATTTGTATTAAATGGCATGATGACAAAAGAACAGTTTCTAGCAGGATACGGCTTAACACAAGCAATTCCAGGTCCTCTTTTTACATTTGCATCTTACATAGGAACGGTACTAGACGGGGCACTAGGAGCTACTATTGCGACAATCGCTATCTTTCTCCCGGCATTCTTACTTGTCATTGGTGTATTACCATTTTGGGATCAAGTGAGACGCTTGTCTTATGTACAAGGGGCACTCCTTGGCATTAATGCCGCCGTCGTCGGTATTTTACTAGCTGCTTTTTATGACCCTATTTGGACAAGCACAATCATGAATAAAACTGATTTTGTTATCGCTTCTCTCTTATTTTGTCTATTAGCATTTTGGAAAGCACCTCCATGGATCGTTGTTGTTCTTGGTGCTTTAAGCGGTTATATTCTTTCTATTATC

**Figure S1. Aminoacid sequence alignment of ChrA from NCr1a to putative ChrA from *B. pseudomycoides* type strain and previously characterized chromate transporters.**

A) The putative chromate transporter ChrA from *B. pseudomycoides* strain NCr1a was compared not only to the ChrA sequence of *B. pseudomycoides* DSM 12442 strain, but also to previously characterized LCHR transporters from both, Gram-positive and Gram-negative species. Blue color corresponds to conservative residues, red to identical residues. The alignment was created by VectorNTi software (Invitrogen), which uses ClustalW tool for multiple sequence alignment. B) The nucleotide sequence of *chrA* cds and its upstream region extracted from *B. pseudomycoides* NCr1a genomic DNA. Start codon is underlined.

**Table S5**. **Identity/Conserved/Distance table of ChrA from NCr1a and characterized chromate transporters**

**A**

| **ChrA from** | NCr1a | *B.pseudomycoides* | *B.cereus SJ1* | *L.fusiformis ZC1* | *O.triticii TnOtChr.* | *P.aeruginosa pUM505* | *S.oneidensis MR-1* | *Shewanella ANA-3* |
| --- | --- | --- | --- | --- | --- | --- | --- | --- |
| NCr1a |  | 99 | 78 | 53 | 24 | 25 | 39 | 41 |
| *B.pseudomycoides* |  |  | 78 | 54 | 24 | 26 | 41 | 42 |
| *B.cereus SJ1* |  |  |  | 54 | 23 | 25 | 40 | 40 |
| *L.fusiformis ZC1.* |  |  |  |  | 23 | 24 | 41 | 40 |
| *O. triticii TnOtChr.* |  |  |  |  |  | 29 | 22 | 22 |
| *P.aeruginosa pUM505* |  |  |  |  |  |  | 26 | 26 |
| *S.oneidensisMR-1* |  |  |  |  |  |  |  | 89 |
| *ShewanellaANA-3* |  |  |  |  |  |  |  |  |

**B**

| **ChrA from** | NCr1a | *B.pseudomycoides* | *B.cereus SJ1* | *L.fusiformis ZC1* | *O.triticii TnOtChr.* | *P.aeruginosa pUM505* | *S.oneidensis MR-1* | *Shewanella ANA-3* |
| --- | --- | --- | --- | --- | --- | --- | --- | --- |
| NCr1a |  | 100 | 90 | 70 | 42 | 47 | 59 | 59 |
| *B.pseudomycoides* |  |  | 90 | 71 | 44 | 47 | 60 | 60 |
| *B.cereus SJ1* |  |  |  | 70 | 42 | 45 | 59 | 58 |
| *L.fusiformis ZC1.* |  |  |  |  | 43 | 46 | 58 | 58 |
| *O. triticii TnOtChr.* |  |  |  |  |  | 46 | 40 | 40 |
| *P.aeruginosa pUM505* |  |  |  |  |  |  | 44 | 45 |
| *S.oneidensisMR-1* |  |  |  |  |  |  |  | 95 |
| *ShewanellaANA-3* |  |  |  |  |  |  |  |  |

**C**

| **ChrA from** | NCr1a | *B.pseudomycoides* | *B.cereus SJ1* | *L.fusiformis ZC1* | *O.triticii TnOtChr.* | *P.aeruginosa pUM505* | *S.oneidensis MR-1* | *Shewanella ANA-3* |
| --- | --- | --- | --- | --- | --- | --- | --- | --- |
| NCr1a |  | 1 | 22 | 47 | 76 | 74 | 61 | 59 |
| *B.pseudomycoides* |  |  | 22 | 46 | 76 | 74 | 59 | 58 |
| *B.cereus SJ1* |  |  |  | 46 | 77 | 75 | 59 | 60 |
| *L.fusiformis ZC1.* |  |  |  |  | 77 | 76 | 59 | 59 |
| *O. triticii TnOtChr.* |  |  |  |  |  | 70 | 78 | 78 |
| *P.aeruginosa pUM505* |  |  |  |  |  |  | 74 | 74 |
| *S.oneidensisMR-1* |  |  |  |  |  |  |  | 11 |
| *ShewanellaANA-3* |  |  |  |  |  |  |  |  |

**D**


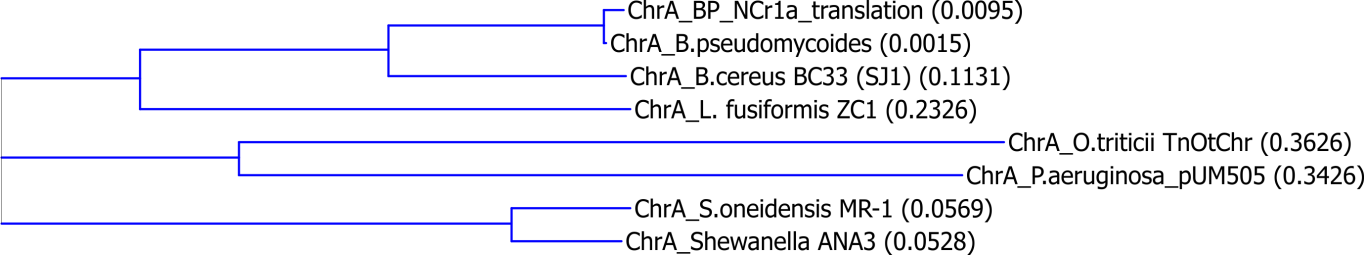


A) Identity table shows the percentage of identical residues among all ungapped positions between the pairs of analysed sequences; B) Conservation table shows the percentage of identical and similar amino acids among all ungapped positions between the pairs of analysed sequences; C) Distance table shows the distance scores between the sequence pairs in percent (which equals 100-identity score). The tables were created in VectorNTi software (Invitrogen). This software uses the Neighbor Joining Method, which works on a matrix of distances between all pairs of sequence analysed. D) Phylogenetic analysis displayed in a treelike diagram (the guide tree) suggesting the evolutionary relationships between the analysed sequences, created by Vector NTI software using the Neighbor Joining method.

The calculated distance values are shown in parenthesis after the sequence name.

**
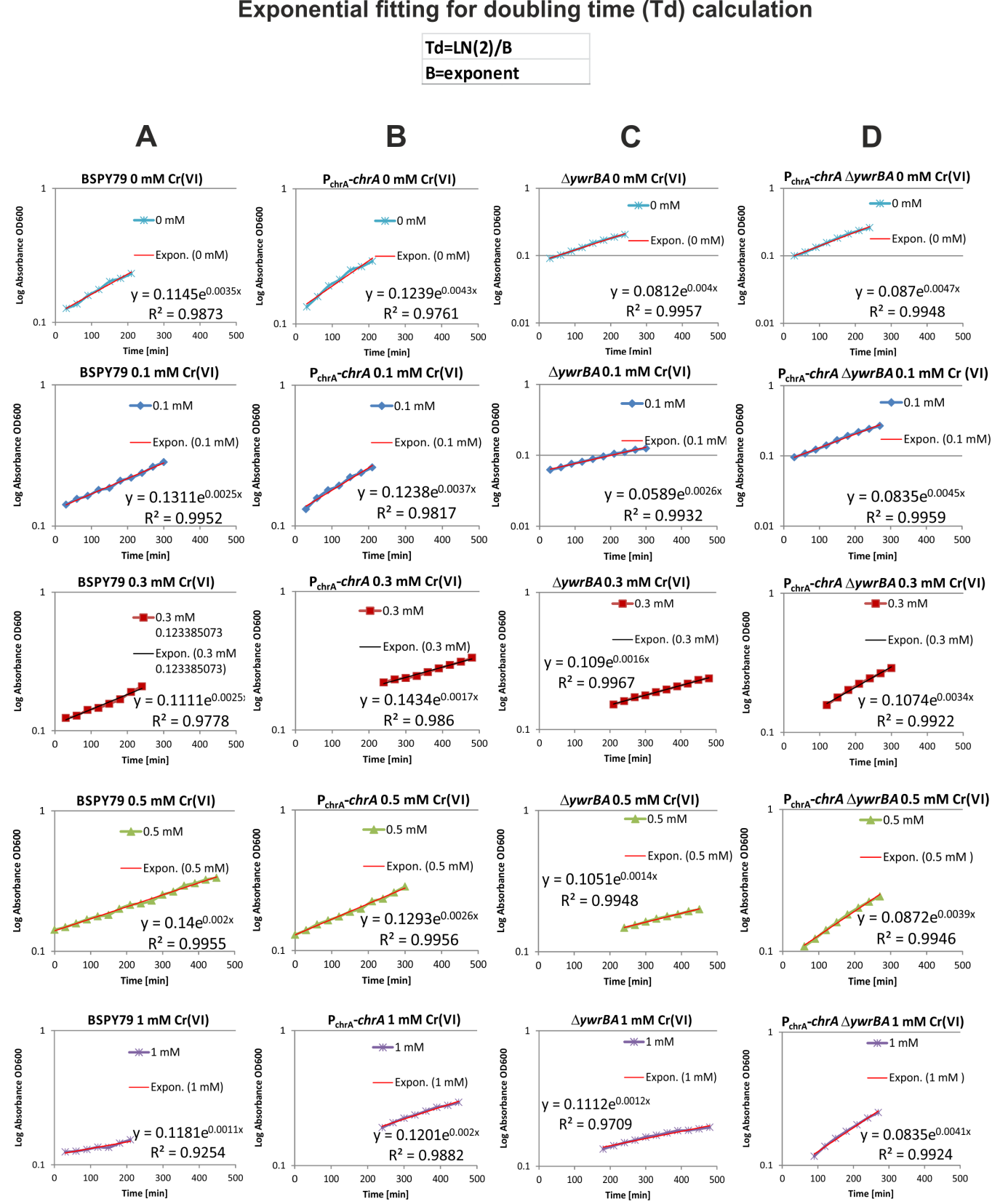
**

**Figure S2. Fitting of growth curves to exponential function**

To calculate the doubling time of each strain in each set of conditions, the “exponent” needs to be estimated. Thus each growth curve is fitted to an exponential function and the best fit (the accuracy of which is indicated by the R^2^ value) is expressed by an equation, from which the exponent is utilized. A-D) The exponential growth of strains in M9 media, at ascending concentrations of Cr(VI).

**Table S6 Estimated doubling times (T_d_) of *B. subtilis* strains**

| **Strain** | | **Cr(VI) concentration** | | | | |
| --- | --- | --- | --- | --- | --- | --- |
|  |  | 0 mM | 0.1 mM | 0.3 mM | 0.5 mM | 1.0 mM |
| PY79 |  | 198.04 | 277.26 | 277.26 | 346.56 | 630.13 |
| P_chrA_-*chrA* | **T_d_** | 161.2 | 187.34 | 407.73 | 266.6 | 346.57 |
| Δ*ywrBA* | [min] | 173.29 | 266.6 | 407.73 | 495.11 | 577.62 |
| P_chrA_-*chrA,* Δ*ywrBA* |  | 147.48 | 154.03 | 203.87 | 177.73 | 169.06 |

**Table S7 Statistical test of differences in doubling times between strains grown under Cr stress**

| SUMMARY | | | | | | | |  |  |  |
| --- | --- | --- | --- | --- | --- | --- | --- | --- | --- | --- |
| **ROWS** | Count | | Sum (T_d_) | Average  (T_d_) | | Variance  (T_d_) | |  |  |  |
| BS PY79 | 5 | | 1729.2 | 345.8 | | 28018.6 | |  |  |  |
| P_chrA_-*chrA* | 5 | | 1369.4 | 273.8 | | 10860.3 | |  |  |  |
| *ΔywrBA* | 5 | | 1920.3 | 384.0 | | 27145.3 | |  |  |  |
| P_chrA_-*chrA*, *ΔywrBA* | 5 | | 852.1 | 170.4 | | 492.2 | |  |  |  |
| **COLUMNS** |  | |  |  | |  | |  |  |  |
| 0 mM Cr(VI) | 4 | | 680.0 | 170.0 | | 460.6 | |  |  |  |
| - 1. mM Cr(VI) | 4 | | 885.2 | 221.3 | | 3620.4 | |  |  |  |
| 0.3 mM Cr(VI) | 4 | | 1296.5 | 324.1 | | 10213.0 | |  |  |  |
| 0.5 mM Cr(VI) | 4 | | 1286.0 | 321.5 | | 18150.5 | |  |  |  |
| 1.0 mM Cr(VI) | 4 | | 1723.3 | 430.8 | | 45630.8 | |  |  |  |
| ANOVA |  |  | | |  | |  | |  |  |
| Source of variation | *SS* | *df* | | | *MS* | | *F* | | *P-value* | *F-crit* |
| Rows | 132367.743 | 3 | | | 44122.581 | | 5.198 | | 0.015695509 | 3.490294819 |
| Columns | 164206.770 | 4 | | | 41051.692 | | 4.836 | | 0.014806082 | 3.259166727 |
| Error | 101859.211 | 12 | | | 8488.267 | |  | |  |  |
|  |  |  | | |  | |  | |  |  |
| Total | 398433.724 | 19 | | |  | |  | |  |  |

Statistical testing of calculated doubling times (T_d_) using Two-Factor Anova without replication (Microsoft Excel 2010) suggests that the source of significant variation are both the genetic background of the strain (i.e. P_chrA_-*chrA*, *ΔywrBA* strain has the lowest variation in doubling time across the tested concentrations of Cr(VI), while *B. subtilis* PY79 exhibits the greatest variation in doubling time); and the concentration of the Cr(VI) in the media (with the exception of P_chrA_-*chrA*, *ΔywrBA* strain, which has the same average T_d_ across all concentrations of Cr(VI) as when grown in media with 0 mM Cr(VI).

**Table S8 Statistical significance of differences in doubling times between the two strains**

| Comparison of Td means between combinations of strains, paired t-test | | | | | |
| --- | --- | --- | --- | --- | --- |
|  | BSPY79 vs  P_chrA_*-chrA* | Δ*ywrBA* vs P_chrA_*chrA*Δ*ywrBA* | BSPY79 vs  P_chrA_*chrA*Δ*ywrBA* | BSPY79 vs  Δ*ywrBA* | Δ*ywrBA* vs  P_chrA_*-chrA* |
| t Stat | 1.088807551 | 4.027010937 | 2.361455763 | -0.910050596 | 2.174916185 |
| P(T<=t) | 0.168722423 | 0.013759507 | 0.038769849 | 0.20713703 | 0.047642293 |
| Significance | No | Significant | Significant | No | Border-significant |
